# Lipid-Lowering Effects of Alpha-Mangostin: A Systematic Review and Meta-Analysis in Hyperlipidemic Animal Models

**DOI:** 10.3390/foods14111880

**Published:** 2025-05-26

**Authors:** Moragot Chatatikun, Aman Tedasen, Phichayut Phinyo, Pakpoom Wongyikul, Passakorn Poolbua, Wiyada Kwanhian Klangbud, Jason C. Huang, Rattana Leelawattana, Atthaphong Phongphithakchai

**Affiliations:** 1 Department of Medical Technology, School of Allied Health Sciences, Walailak University, Nakhon Si Thammarat 80160, Thailand; moragot.ch@wu.ac.th (M.C.); aman.te@wu.ac.th (A.T.); patsakorn.po@mail.wu.ac.th (P.P.); 2 Research Excellence Center for Innovation and Health Products (RECIHP), Walailak University, Nakhon Si Thammarat 80160, Thailand; 3 Center for Clinical Epidemiology and Clinical Statistics, Faculty of Medicine, Chiang Mai University, Chiang Mai 50200, Thailand; phichayut.phinyo@cmu.ac.th (P.P.); aumkidify@gmail.com (P.W.); 4 Department of Biomedical Informatics and Clinical Epidemiology (BioCE), Faculty of Medicine, Chiang Mai University, Chiang Mai 50200, Thailand; 5 Medical Technology Program, Faculty of Science, Nakhon Phanom University, Nakhon Phanom 48000, Thailand; wiyadakwanhian@gmail.com; 6 Department of Biotechnology and Laboratory Science in Medicine, National Yang Ming Chiao Tung University, Taipei 112304, Taiwan; jasonhuang@nycu.edu.tw; 7 Endocrinology and Metabolism Unit, Division of Internal Medicine, Faculty of Medicine, Prince of Songkla University, Songkhla 90110, Thailand; lrattana@meicine.psu.ac.th; 8 Nephrology Unit, Division of Internal Medicine, Faculty of Medicine, Prince of Songkla University, Songkhla 90110, Thailand

**Keywords:** alpha-mangostin, hyperlipidemia, animal model, triglyceride, cholesterol, LDL-C, HDL-C

## Abstract

Hyperlipidemia is a major risk factor for cardiovascular and metabolic diseases. Although pharmacologic treatments are effective, their adverse effects have spurred interest in natural alternatives. Alpha-mangostin (AM), a xanthone from *Garcinia mangostana*, has shown lipid-lowering effects in animal studies, but its overall efficacy remains unclear. This systematic review and meta-analysis, conducted in accordance with PRISMA 2020 guidelines, evaluated AM’s impact on lipid profiles in hyperlipidemic animal models. Databases including Scopus, PubMed, ScienceDirect, Cochrane Library, and Web of Science were searched for relevant controlled studies. Nine studies (N = 226 animals) met inclusion criteria, reporting data on triglycerides (TG), total cholesterol (TC), LDL-C, and HDL-C. Risk of bias, assessed using the Cochrane RoB 2 tool, was generally low-to-moderate. Meta-analysis using a random-effects model revealed that AM significantly reduced TG, TC, and LDL-C, while increasing HDL-C. Stronger effects were observed at doses <50 mg/kg/day. Subgroup and sensitivity analyses confirmed robustness and highlighted the influence of species, region, and treatment duration. These findings suggest that AM is a promising lipid-lowering agent in animal models. Further clinical trials are needed to validate efficacy in humans and determine optimal dosing.

## 1. Introduction

Hyperlipidemia, characterized by elevated levels of total cholesterol (TC), triglycerides (TG), and low-density lipoprotein cholesterol (LDL-C), along with reduced high-density lipoprotein cholesterol (HDL-C), is a major risk factor for cardiovascular diseases (CVDs), stroke, and type 2 diabetes [1]. It contributes significantly to the global burden of non-communicable diseases (NCDs) and is commonly associated with metabolic syndrome and obesity [2]. Although statins, fibrates, and other pharmacologic agents are widely used to manage dyslipidemia, they are often accompanied by adverse effects such as myopathy, elevated liver enzymes, and glucose intolerance [3,4]. Consequently, there is growing interest in natural compounds with lipid-lowering potential and minimal side effects.

**Figure 1:**
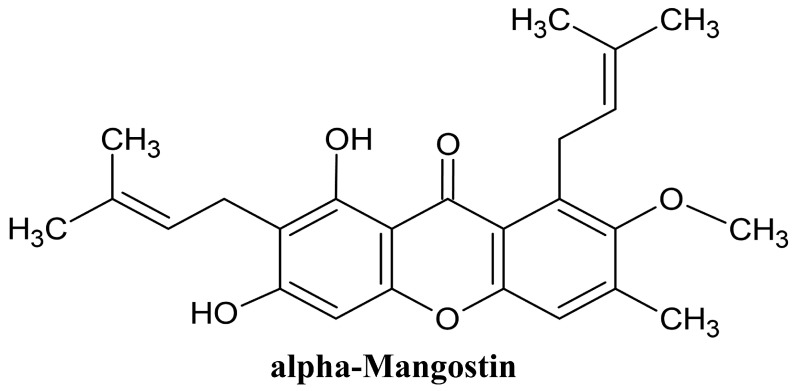
Chemical structure of alpha-mangostin (AM).

One such compound is alpha-mangostin, a bioactive xanthone derived predominantly from the pericarp of *Garcinia mangostana* L. (commonly known as mangosteen), a tropical fruit native to Southeast Asia [5]. It has been reported to exhibit a wide range of biological activities, including antioxidant, anti-inflammatory, antimicrobial, anticancer, anti-obesity, and lipid-lowering effects [6,7,8,9]. Alpha-mangostin (AM) is a prenylated xanthone characterized by a tricyclic aromatic xanthone core with hydroxyl groups at C-1, C-3, and C-6 and lipophilic prenyl side chains at C-2 and C-8, which enhance its membrane permeability and bioactivity, as shown in Figure 1 [10,11].

Pharmacokinetic studies indicate that AM is rapidly absorbed but undergoes extensive metabolism, particularly glucuronidation and sulfation in the liver, which may limit its bioavailability at higher doses [6,12]. Despite this, AM’s lipophilicity facilitates tissue distribution, especially in lipid-rich organs such as the liver and adipose tissue—key sites of lipid metabolism [6]. The major metabolic products detected in vivo include alpha-mangostin-3-O-glucuronide and alpha-mangostin-6-O-sulfate, which are excreted through bile and urine [12]. These conjugated metabolites may still retain biological activity and have been implicated in antioxidant and anti-inflammatory effects [6]. Mechanistically, AM exerts lipid-lowering effects primarily through activation of AMP-activated protein kinase (AMPK) and sirtuin-1 (SIRT1), inhibition of SREBP-1c and fatty acid synthase (FAS), and enhancement of cholesterol efflux via ATP-binding cassette transporter A1 (ABCA1) [13,14,15]. While its main metabolites, alpha-mangostin-3-O-glucuronide and 6-O-sulfate, are less well-characterized, they may retain residual activity or serve as transport forms contributing to its overall metabolic effects [12]. Its anti-inflammatory properties also contribute to its metabolic benefits by suppressing pro-inflammatory cytokines (IL-6, TNF-α) and inhibiting the NF-κB and TLR4 signaling pathways, which are often upregulated in dyslipidemia and insulin resistance [16,17]. These effects collectively lead to suppression of lipid synthesis, increased fatty acid oxidation, and improved lipid profile regulation.

Although several in vivo studies have explored the hypolipidemic properties of AM, the findings vary considerably due to differences in animal models, induction methods, doses, and durations of treatment. Moreover, no prior meta-analysis has comprehensively synthesized the available evidence to quantify its lipid-lowering effects or evaluate dose-dependent responses. Notably, a recent systematic review and network meta-analysis highlighted the hypoglycemic activity of *Garcinia mangostana* L. extracts in diabetic rodent models, demonstrating significant reductions in fasting blood glucose and improvements in lipid profiles [18]. Therefore, this study aimed to conduct a systematic review and meta-analysis of animal studies to determine the effect of AM on serum lipid profiles, including TG, TC, LDL-C, and HDL-C. In addition, we evaluated potential dose-related patterns and assessed study quality to provide a clearer understanding of AM’s therapeutic value in hyperlipidemia.

## 2. Materials and Methods

### 2.1. Study Registration

This systematic review and meta-analysis was registered in the International Prospective Register of Systematic Reviews (PROSPERO) under registration number CRD42024523082. The study protocol was developed in accordance with the Preferred Reporting Items for Systematic Reviews and Meta-Analyses (PRISMA) guidelines to ensure transparency and reproducibility [19]. The PRISMA checklist is provided in Supplementary Table S1.

### 2.2. Literature Search Strategy

A comprehensive literature search was conducted to identify relevant research articles assessing the effect of AM on lipid profiles. The search was performed across the following electronic databases: Scopus, ScienceDirect, PubMed, the Cochrane Library, and Web of Science, covering all available studies up to November 2024. The search terms, detailed in Supplementary Tables S2–S6, were based on keywords related to AM and hyperlipidemia. The search was restricted to articles published in English.

### 2.3. Study Selection

After excluding duplicate studies, two independent investigators (M.C. and A.P.) screened titles and abstracts for eligibility. Original studies were included in the present meta-analysis if they met the following criteria: (1) experimental animal studies with control groups; (2) studies involving animal models with induced hyperlipidemia or naturally occurring elevated lipid profiles; (3) studies administering AM to evaluate its effects on lipid profiles, regardless of dosage or route of administration; (4) studies with vehicle- or placebo-treated control groups; (5) studies reporting quantitative data on lipid profiles, including triglycerides (TG), total cholesterol (TC), low-density lipoprotein cholesterol (LDL-C), and high-density lipoprotein cholesterol (HDL-C); and (6) articles published in English.

Studies were excluded if they met any of the following criteria: (1) in vitro studies, review articles, conference abstracts, case reports, or studies without a control group; (2) studies using healthy animals without hyperlipidemia or elevated lipid profiles; (3) studies investigating AM in combination with other herbal extracts or chemicals; (4) studies lacking a clearly defined control or comparator group; (5) studies that did not report relevant lipid profile data; or (6) non-English articles. Table 1 outlines the PICO (Participants, Intervention/Exposure, Comparison, Outcomes, and Study Design) criteria used to define the research question.

### 2.4. Data Extraction

Publications and all data from eligible studies were independently screened and extracted by two authors (M.C. and A.P.). Any discrepancies were resolved through discussion or consultation with a third reviewer (A.T.). The following data were extracted: the first author’s last name, year of publication, animal species, hyperlipidemia induction model, dosage of AM administration, study region, treatment period, and lipid profile parameters (TG, TC, LDL-C, HDL-C).

In cases where information in the included studies was unclear, clarification was sought by emailing the corresponding authors. Furthermore, when lipid profile results were reported in mmol/L, we used the OnlineConversion.com electronic calculator to convert these values to mg/dL, adjusting as necessary based on the type of cholesterol measured. For continuous outcome measures, the mean and standard deviation (SD) of the results, along with the number of hyperlipidemia models, were collected. If studies reported the standard errors of the mean (SEM) instead of SDs, the SDs were calculated by multiplying the SEM by the square root of the corresponding sample size. When necessary, SDs were imputed following the guidelines outlined in the *Cochrane Handbook for Systematic Reviews of Interventions* [20].

### 2.5. Quality Assessment

The Risk-of-Bias 2 (RoB 2) assessment tool developed by the Cochrane Collaboration was applied to evaluate potential risks of bias in the included studies [21]. Each study was assessed for domain-specific quality across five aspects: bias arising from the randomization process; bias due to deviations from intended interventions; bias due to missing outcome data; bias in measurement of the outcome; and bias in selection of the reported result. Studies were categorized as having a low risk of bias, high risk of bias, or some concerns in each domain.

### 2.6. Data Synthesis and Analysis

The studies selected for meta-analysis included the following outcomes: TG, TC, LDL-C, and HDL-C. Both baseline and post-treatment data from control and treatment groups in preclinical studies were analyzed. All statistical analyses were performed using Stata version 17 (StataCorp LLC, College Station, TX, USA). Heterogeneity among studies was assessed using Cochran’s Q test and the *I*^2^ statistic, with heterogeneity classified as low (0–25%), moderate (26–50%), substantial (51–75%), or considerable (*I*^2^ > 75%) [22]. Continuous data were standardized to the same scale, and treatment effects were summarized using mean differences (MDs) with 95% confidence intervals (CIs) [23]. A random-effects model was applied to account for potential variability among studies [24].

Subgroup analyses were performed in cases of high heterogeneity (*I*^2^ > 50%) to identify potential sources of variability based on differences in animal species, study region, and experimental duration. Sensitivity analyses were conducted by excluding studies with imputed data or those identified as having a high risk of bias, in order to evaluate the robustness of the primary findings [25]. Publication bias was planned to be assessed through visual inspection of funnel plots and statistical methods such as Egger’s regression test, which is commonly used to detect asymmetry that may indicate bias due to small-study effects [26]. However, as the number of included studies in the meta-analysis was fewer than 10, this method was not applied [27].

## 3. Results

### 3.1. Literature Search

**Figure 2:**
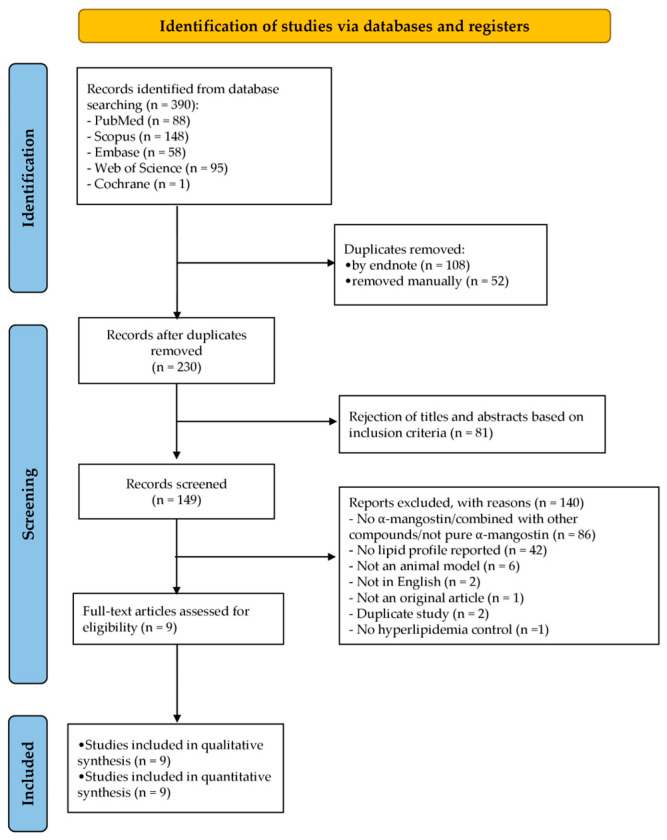
Flowchart illustrating the search strategy and study selection process in accordance with the PRISMA 2020 (Preferred Reporting Items for Systematic Reviews and Meta-Analyses) guidelines.

A total of 390 records were identified through database searches, including PubMed (88), Scopus (148), Embase (58), Web of Science (95), and Cochrane Library (1). After removing 160 duplicate records (108 through EndNote and 52 manually), 230 studies remained for screening. Following title and abstract screening, 81 records were excluded based on the inclusion criteria, leaving 149 studies for further assessment. Full-text evaluation was conducted for 9 studies, while 140 studies were excluded for the following reasons: absence of pure AM or its combination with other compounds (*n* = 86), lack of lipid profile data (*n* = 42), non-animal model studies (*n* = 6), non-English publications (*n* = 2), non-original articles (*n* = 1), duplicated studies (*n* = 2), and absence of a hyperlipidemia control group (*n* = 1). Ultimately, nine studies met the inclusion criteria and were included in both the qualitative synthesis and quantitative meta-analysis, focusing on the lipid-lowering effects of AM in hyperlipidemic animal models, as illustrated in Figure 2.

### 3.2. Study Characteristics

A total of nine preclinical studies were included in this systematic review and meta-analysis, conducted across five countries: China (*n* = 3) [17,28,29], South Korea (*n* = 2) [13,30], Indonesia (*n* = 2) [15,31], Iran (*n* = 1) [32], and Thailand (*n* = 1) [33], as shown in Table 2. The studies utilized various animal species, including Wistar rats (*n* = 3), Sprague-Dawley rats (*n* = 3), C57BL/6 mice (*n* = 2), C57BL/KsJ-diabetic (db/db) mice (*n* = 1), and Swiss albino Wistar rats (*n* = 1). These studies employed different experimental models, including high-fat diet-induced obesity (*n* = 2), diabetes mellitus (*n* = 3), streptozotocin-induced gestational diabetes (*n* = 1), insulin resistance (*n* = 1), olanzapine-induced metabolic disorders (*n* = 1), and adjuvant-induced arthritis (*n* = 1). AM was primarily administered orally (*n* = 7), while two studies utilized intraperitoneal injection (*n* = 2). The treatment durations were categorized as <8 weeks (*n* = 4) and ≥8 weeks (*n* = 5). Dosages of AM varied across the studies, ranging from 5 mg/kg/day to 200 mg/kg/day, with some studies investigating multiple dose levels, such as Ardakanian et al. 2022 administering 10, 20, and 40 mg/kg/day, and Soetikno et al. 2022, 2033 using 100 and 200 mg/kg/day [15,31,32]. All studies assessed changes in total cholesterol (TC) and triglycerides (TG), with six studies also measuring low-density lipoprotein cholesterol (LDL-C) and high-density lipoprotein cholesterol (HDL-C). The total number of animals included in the treatment and control groups across all studies was N = 226, with group sizes ranging from (6/6) to (10/10) per study.

### 3.3. Risk-of-Bias Assessment

**Figure 3:**
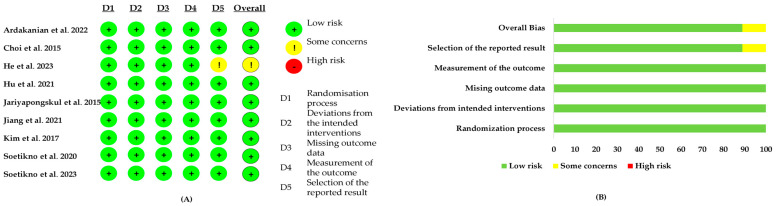
Risk-of-bias assessment using the RoB 2 tool for the included preclinical studies evaluating the lipid-lowering effects of alpha-mangostin: (**A**) Dot plot displaying the risk−of−bias judgments for each study across five domains: D1 = Randomization process, D2 = Deviations from intended interventions, D3 = Missing outcome data, D4 = Measurement of the outcome, and D5 = Selection of the reported result. (**B**) Bar chart summarizing the overall proportion of studies rated as low risk (green), some concerns (yellow), or high risk (red) in each domain. Most studies were assessed as having a low risk of bias, with minor concerns observed in the selection of reported results [13,15,17,28,29,30,31,32,33].

The risk-of-bias assessment using the RoB 2 tool indicated that the overall methodological quality of the included studies was generally high, as shown in Figure 3A,B. Most domains, including the randomization process (D1), deviations from intended interventions (D2), missing outcome data (D3), and measurement of the outcome (D4), were judged to have a low risk of bias across all studies. However, some concerns were noted in the domain of selection of the reported result (D5), suggesting the possibility of selective outcome reporting in a few studies. As a result, the overall bias was also rated as having some concerns in a similar proportion of studies. Notably, none of the studies were classified as having a high risk of bias in any domain, indicating that the evidence base is largely reliable with minimal potential for bias.

### 3.4. Result of Meta-Analysis

#### 3.4.1. Effect of AM on Triglyceride (TG)

**Figure 4:**
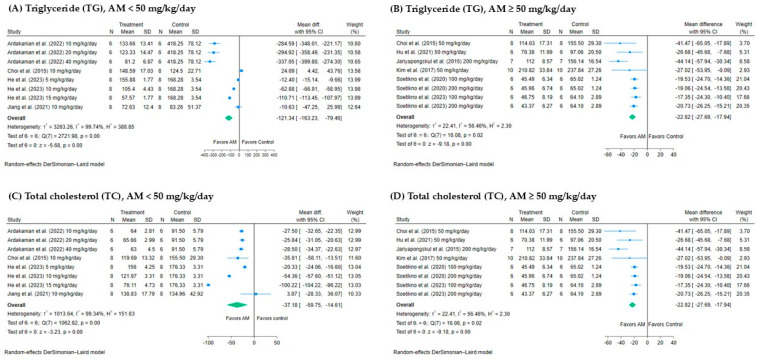
Forest plots showing the effects of alpha-mangostin (AM) on triglyceride (TG) and total cholesterol (TC) levels at different doses: (**A**) TG, AM <50 mg/kg/day. (**B**) TG, AM ≥50 mg/kg/day. (**C**) TC, AM <50 mg/kg/day. (**D**) TC, AM ≥50 mg/kg/day. The x-axis represents the mean difference in TG and TC (mg/dL) between the treatment and control groups. Each horizontal line indicates the 95% confidence interval (CI) for an individual study, with the square representing the effect size and its size proportional to the study’s weight. The diamond at the bottom of each plot represents the pooled effect estimate and its CI, calculated using the random-effects DerSimonian–Laird model [13,15,17,28,29,30,31,32,33]. SD: standard deviation of the differences.

The meta-analysis evaluated the effect of AM on TG levels at different dosage ranges using a random-effects DerSimonian–Laird model as shown in Figure 4A,B. For studies using AM doses <50 mg/kg/day, the pooled mean difference was markedly greater at −121.34 mg/dL (95% CI: −163.23 to −79.46), also statistically significant (*p* < 0.001), but with considerable heterogeneity (*I*^2^ = 99.74%), suggesting considerable variability between studies. In contrast, AM doses ≥50 mg/kg/day, the pooled mean difference in TG levels between treatment and control groups was −22.82 mg/dL (95% CI: −27.69 to −17.94), indicating a statistically significant reduction in TG levels favoring the AM group, with substantial heterogeneity (*I*^2^ = 56.46%, *p* < 0.001). Both dosage ranges demonstrated TG-lowering effects of AM; however, the greater magnitude of reduction at lower doses, coupled with considerable heterogeneity, suggests dose-dependent variability and highlights the need for further investigation into contributing factors such as animal species, sources, and treatment period.

#### 3.4.2. Effect of AM on Total Cholesterol (TC)

A total of nine studies were included in the meta-analysis to assess the effect of AM on TC levels, stratified by dosage into two groups: six studies with AM doses ≥50 mg/kg/day and four studies with doses <50 mg/kg/day, as shown in Figure 4C,D. For studies administering AM <50 mg/kg/day showed a larger pooled mean difference of −37.18 mg/dL (95% CI: −59.75 to −14.61), also statistically significant, but with considerable heterogeneity (*I*^2^ = 99.34%, *p* value < 0.001), indicating substantial variability across the studies. While for AM ≥ 50 mg/kg/day, the pooled mean difference was −22.82 mg/dL with a 95% confidence interval of −27.69 to −17.94, indicating a statistically significant reduction in TC levels in the AM-treated groups compared to control. The heterogeneity was substantial (*I*^2^ = 56.46%, *p* value < 0.001), suggesting some variability among the studies. Both dosage ranges support the TC-lowering effect of AM, though the greater heterogeneity at lower doses highlights the need for cautious interpretation and further exploration of animal species, study region, and treatment duration.

#### 3.4.3. Effect of AM on Low-Density Lipoprotein Cholesterol (LDL-C)

**Figure 5:**
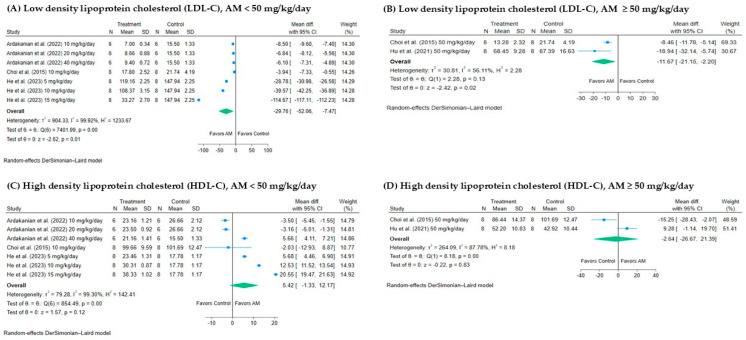
Forest plots showing the effect of alpha-mangostin (AM) on low-density lipoprotein cholesterol (LDL-C) and high-density lipoprotein cholesterol (HDL-C) levels at different doses: (**A**) LDL-C, AM < 50 mg/kg/day. (**B**) LDL-C, AM ≥ 50 mg/kg/day. (**C**) HDL-C, AM < 50 mg/kg/day. (**D**) HDL-C, AM ≥ 50 mg/kg/day. The x-axis represents the mean difference in LDL-C and HDL-C (mg/dL) between the treatment and control groups. Each horizontal line indicates the 95% confidence interval (CI) for an individual study, with the square representing the effect size, sized proportionally to the study’s weight. The diamond at the bottom of each plot represents the pooled effect estimate and its CI, calculated using the random-effects DerSimonian–Laird model [13,17,28,32].

The meta-analysis assessed the effect of AM on LDL-C levels at different dosage ranges, as shown in Figure 5A,B. For studies using AM <50 mg/kg/day (Figure 5A), the pooled mean difference was −29.76 mg/dL (95% CI: −52.06 to −7.47), indicating a statistically significant reduction in LDL-C levels in the AM-treated group compared to the control. The heterogeneity was considerable (*I*^2^ = 99.92%, *p* value = 0.01), suggesting variability among the studies. In contrast, for studies using AM ≥ 50 mg/kg/day (Figure 5B), the pooled mean difference was −11.67 mg/dL (95% CI: −21.15 to −2.20), also statistically significant, with lower heterogeneity (*I*^2^ = 56.11%, *p* value = 0.02). Both dosage groups showed a significant reduction in LDL-C, with AM < 50 mg/kg/day demonstrating a slightly greater effect, although the higher heterogeneity in the lower dose range indicates some variability in study results.

#### 3.4.4. Effect of AM on High-Density Lipoprotein Cholesterol (HDL-C)

The meta-analysis evaluated the effect of AM on HDL-C levels at two different dosage ranges, as presented in Figure 5C,D. For studies using AM < 50 mg/kg/day (Figure 4C), the pooled mean difference was 5.42 mg/dL (95% CI: −1.33 to 12.17), indicating a statistically significant increase in HDL-C levels in the AM-treated group compared to the control. Heterogeneity was high (*I*^2^ = 99.30%, *p* < 0.001), suggesting considerable variability among the studies. In contrast, for studies using AM ≥ 50 mg/kg/day (Figure 5D), the pooled mean difference was −2.64 mg/dL (95% CI: −26.67 to 21.39), which was not statistically significant, with lower heterogeneity (*I*^2^ = 87.78%, *p* = 0.83). Both dosage groups demonstrated an increase in HDL-C levels, with the <50 mg/kg/day group showing a greater effect. However, the higher heterogeneity observed in this group suggests that further studies are needed to explore potential sources of variability.

### 3.5. Subgroup Analysis

#### 3.5.1. Subgroup Analysis of the Effect of AM on TG Levels

**Figure 6:**
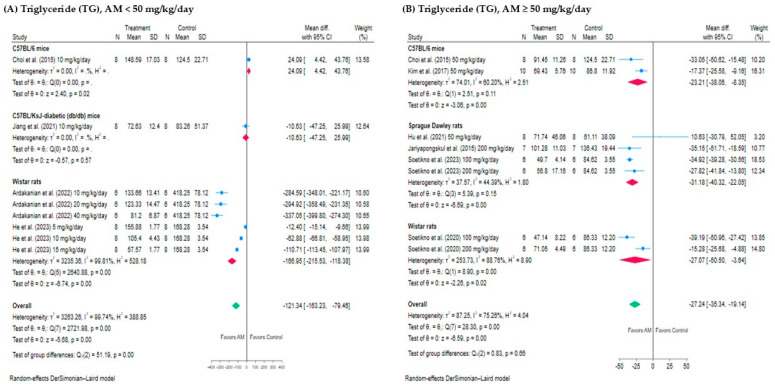
Subgroup analysis of the effect of alpha-mangostin (AM) on triglyceride (TG) levels stratified by species: (**A**) AM < 50 mg/kg/day: subgrouped by species (C57BL/6 mice, C57BL/KsJ diabetic (db/db) mice, and Wistar rats); (**B**) AM ≥ 50 mg/kg/day: subgrouped by species (C57BL/6 mice, Sprague-Dawley rats, and Wistar rats). Each subgroup presents the mean difference in TG levels (mg/dL) between the AM-treated and control groups with corresponding 95% confidence intervals (CIs). Squares indicate study effect sizes with size proportional to weight; horizontal lines show 95% CIs; diamonds represent pooled effect sizes with 95% CIs. Heterogeneity statistics (*I*^2^ and *H*^2^) were not reported for subgroups that included only a single study, as these measures required a minimum of two studies to assess variability between results. The analysis was conducted using a random-effects model [13,15,17,28,29,31,32,33].

The subgroup analysis assessed the effect of AM on TG levels across different species, countries, and treatment durations. For AM < 50 mg/kg/day, the analysis of C57BL/6 mice and C57BL/KsJ diabetic (db/db) mice showed limited generalizability due to only one study per species, as shown in Figure 6A. In Wistar rats, significant reductions in TG levels were observed (−166.95 mg/dL, 95% CI: −215.53 to −118.38, *I*^2^ = 99.81%, *p* < 0.001). For AM ≥ 50 mg/kg/day, significant reductions in TG levels were seen in C57BL/6 mice was −23.21 mg/dL (95% CI: −38.06 to −8.35, *I*^2^ = 60.20%, *p* < 0.001), Sprague-Dawley rats (−31.18 mg/dL, 95% CI: −40.32 to −22.05, *I*^2^ = 44.39%, *p* < 0.001), and Wistar rats (−27.07 mg/dL, 95% CI: −50.50 to −3.64, *I*^2^ = 88.76%, *p* = 0.02), as shown in Figure 6B.

**Figure 7:**
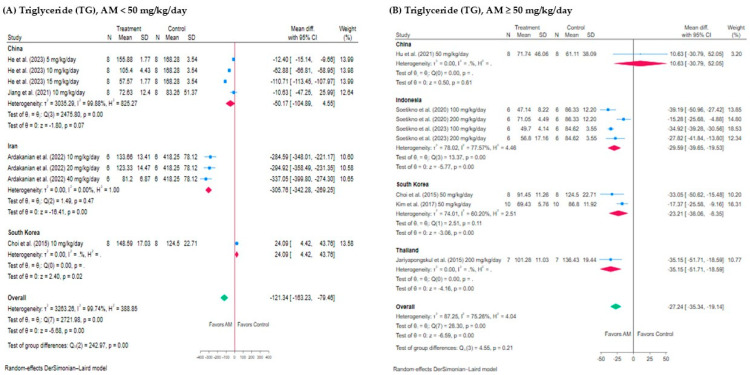
Subgroup analysis of the effect of alpha-mangostin (AM) on triglyceride (TG) levels by country: (**A**) AM < 50 mg/kg/day: studies conducted in China, South Korea, and Iran. (**B**) AM ≥ 50 mg/kg/day: studies conducted in China, Indonesia, South Korea, and Thailand. Each subgroup presents the mean difference in TG levels (mg/dL) between AM-treated and control groups with corresponding 95% confidence intervals (CIs). Squares indicate study effect sizes with size proportional to weight; horizontal lines show 95% CIs; diamonds represent pooled effect sizes with 95% CIs. Heterogeneity statistics (*I*^2^ and *H*^2^) were not reported for subgroups that included only a single study, as these measures required a minimum of two studies to assess variability between results. Analysis was performed using a random-effects model to account for between-study heterogeneity [13,15,17,28,29,30,31,32,33].

In the subgroup of studies conducted in China, alpha-mangostin (AM) showed a mean reduction with no statistically significant change in TG levels (−50.17 mg/dL, 95% CI: −104.89 to 4.55, *I*^2^ = 99.88%, *p* = 0.07). In the Iran subgroup, AM was associated with a large reduction in TG levels (−305.76 mg/dL; 95% CI: −342.88 to −269.25), but this finding is based on a single study. In the South Korea subgroup, AM was associated with an increase in triglyceride (TG) levels of 24.09 mg/dL (95% CI: 4.42 to 43.76), and the result was statistically significant (*p* = 0.02), as shown in Figure 7A. For AM ≥ 50 mg/kg/day, significant reductions were observed in Indonesia (−29.59 mg/dL, 95% CI: −39.65 to −19.53, *I*^2^ = 77.57%, *p* < 0.001) and South Korea (−23.21 mg/dL, 95% CI: −38.06 to −8.35, *I*^2^ = 60.20%), but China showed no significant reduction (*p* = 0.61), as shown in Figure 7B. High heterogeneity was observed across most subgroups (*I*^2^ values above 75%), particularly in China and Indonesia, suggesting variability in responses to AM.

**Figure 8:**
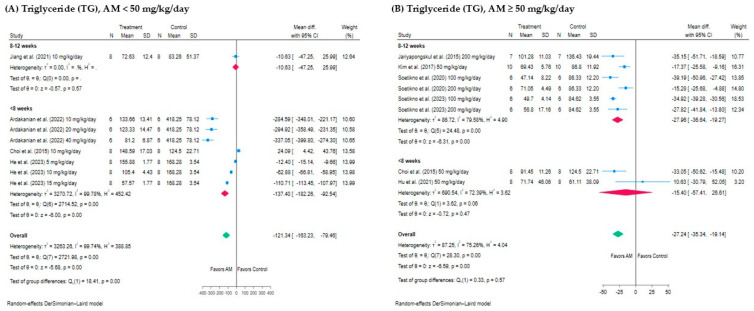
Subgroup analysis of the effect of AM on triglyceride (TG) levels stratified by treatment duration: (**A**) Studies using AM < 50 mg/kg/day categorized by treatment durations of <8 weeks and 8–12 weeks. (**B**) Studies using AM ≥ 50 mg/kg/day categorized by treatment durations of <8 weeks and 8–12 weeks. Each forest plot displays the mean difference in TG levels (mg/dL) between AM-treated and control groups with 95% confidence intervals (CIs). Squares indicate study effect sizes with size proportional to weight; horizontal lines show 95% CIs; diamonds represent pooled effect sizes with 95% CIs. Heterogeneity statistics (*I*^2^ and *H*^2^) were not reported for subgroups that included only a single study, as these measures required a minimum of two studies to assess variability between results. Subgroup heterogeneity and overall effects were assessed using a random-effects model [13,17,28,29,30,32,33].

In the subgroup analysis of treatment durations for AM effects on TG levels, the analysis was divided into two time periods: 8–12 weeks and <8 weeks. For AM < 50 mg/kg/day, the analysis by treatment duration showed no significant change in 8–12 weeks (−10.63 mg/dL, 95% CI: −47.25 to 25.99, *p* = 0.57), but a significant reduction in <8 weeks (−137.40 mg/dL, 95% CI: −182.26 to −92.54, *p* < 0.001), as shown in Figure 8A. For AM ≥ 50 mg/kg/day, a significant reduction was seen in 8–12 weeks (−27.96 mg/dL, 95% CI: −36.64 to −19.27, *p* < 0.001), but no significant effect was found for <8 weeks (−15.40 mg/dL, 95% CI: −57.41 to 26.61, *p* = 0.47), as shown in Figure 8B. Overall, for both dosages, the longer treatment duration of 8–12 weeks was associated with more consistent results in reducing triglyceride levels, while the shorter duration (<8 weeks) showed stronger effects for AM < 50 mg/kg/day but not for AM ≥ 50 mg/kg/day.

#### 3.5.2. Subgroup Analysis of the Effect of AM on TC Levels

The subgroup analysis in Supplementary Figure S1A evaluated the effect of AM < 50 mg/kg/day on TC levels across animal species. For AM < 50 mg/kg/day, significant reductions in TC were observed in C57BL/6 mice (−35.81 mg/dL, *p* < 0.001) and Wistar rats (−42.82 mg/dL, *p* < 0.001), but no significant change was found in C57BL/KsJ-diabetic (db/db) mice (3.87 mg/dL, 95% CI: −28.33 to 36.07, *p* = 0.81). For AM ≥ 50 mg/kg/day (Figure S2B), C57BL/6 mice showed a significant reduction (−35.20 mg/dL, 95% CI: −52.94 to −17.46, *p* < 0.001), as did Sprague-Dawley rats (−25.48 mg/dL, 95% CI: −35.06 to −15.09, *p* = 0.01, *I*^2^ = 75.08%), although with considerable heterogeneity, and Wistar rats also showed a significant reduction (−19.31 mg/dL, 95% CI: −23.07 to −15.55, *p* < 0.001) as shown in Supplementary Figure S1B. Overall, AM consistently reduced TC levels across species and dosages, but the considerable heterogeneity, particularly in studies with fewer samples or single studies per species, suggests the need for further large-scale studies to confirm these findings.

The subgroup analysis of AM’s effect on TC levels across various countries is shown in Supplementary Figure S2A,B. In China, for AM < 50 mg/kg/day, significant reductions were observed in two studies with varying dosages, showing a pooled mean difference of −44.66 mg/dL (95% CI: −82.47 to −6.85, *I*^2^ = 99.64%, *p* = 0.02). Similarly, for AM ≥ 50 mg/kg/day in China, a significant reduction was noted with a pooled mean difference of −26.68 mg/dL (95% CI: −45.68 to −7.68, *p* = 0.01). In Iran, a single study showed a significant reduction for AM < 50 mg/kg/day (−27.19 mg/dL, 95% CI: −30.30 to −24.08, *p* < 0.001. In Indonesia, a single study found significant reductions in TC levels for AM ≥ 50 mg/kg/day, with a pooled mean difference of −19.36 mg/dL (95% CI: −22.20 to −16.52, *p* < 0.001). South Korea showed a significant reduction for both AM < 50 mg/kg/day (−35.81 mg/dL, *p* < 0.001) and AM ≥ 50 mg/kg/day, with a pooled mean difference of −35.20 mg/dL (95% CI: −52.94 to −17.46, *I*^2^ = 0%, *p* < 0.001) and low heterogeneity. In Thailand, a single study demonstrated a significant reduction for AM ≥ 50 mg/kg/day, with a mean difference of −44.14 mg/dL (95% CI: −57.94 to −30.34, *p* < 0.001).

The subgroup analysis examined the effect of AM on TC levels, categorized by treatment durations of AM < 50 mg/kg/day and AM ≥ 50 mg/kg/day, as shown in Supplementary Figure S3A,B. For AM < 50 mg/kg/day, in the 8–12 weeks subgroup, a single study in China showed no significant change in total cholesterol levels, with a pooled mean difference of −3.87 mg/dL (95% CI: −28.33 to 36.07, *p* = 0.81). In contrast, for treatment durations of <8 weeks, significant reductions were observed across multiple studies, with a pooled mean difference of −41.91 mg/dL (95% CI: −65.70 to −18.12, *I*^2^ = 99.43%, *p* < 0.001). For AM ≥ 50 mg/kg/day, the results were similar in terms of significant reductions in cholesterol levels. In the 8–12 weeks subgroup, the pooled mean difference was −21.73 mg/dL (95% CI: −26.65 to −16.81, *p* < 0.001). For treatment durations of <8 weeks, the subgroup showed an overall pooled mean difference of −32.50 mg/dL (95% CI: −47.30 to −17.71, *p* < 0.001). Heterogeneity was considerable, particularly for the shorter treatment durations and AM < 50 mg/kg/day, suggesting a need for more consistent data from future studies to better assess the effect of AM on total cholesterol levels.

#### 3.5.3. Subgroup Analysis of the Effect of AM on LDL-C Levels

The subgroup analysis assessed the effect of AM on LDL-C levels in different species, as shown in Supplementary Figure S4A,B. In C57BL/6 mice, only one study was included in each subgroup for both AM < 50 mg/kg/day and AM ≥ 50 mg/kg/day. For AM < 50 mg/kg/day in Wistar rats, the pooled mean difference was −34.06 mg/dL (95% CI: −58.65 to −9.47, *I*^2^ = 99.79%, *p* = 0.01), indicating a significant reduction in LDL-C levels with considerable heterogeneity. For AM ≥ 50 mg/kg/day, one study was included in each dosage subgroup for both Sprague-Dawley rats and C57BL/6 mice. Overall, all species showed significant reductions in LDL-C levels across both dosages, although the limited number of studies for each subgroup limits the generalizability of the findings.

The subgroup analysis of AM’s effect on LDL-C levels across countries, each represented by a single study, demonstrated consistent LDL-C reductions in all included nations. At doses < 50 mg/kg/day, significant LDL-C decreases were observed in China (−61.01 mg/dL; 95% CI: −114.76 to −7.25), Iran (−7.17 mg/dL; 95% CI: −8.62 to −5.72), and South Korea (−3.94 mg/dL; 95% CI: −7.33 to −0.55), as shown in Supplementary Figure S5A,B. At doses ≥ 50 mg/kg/day, China and South Korea also showed meaningful reductions of −18.94 mg/dL (95% CI: −32.14 to −5.74) and −8.46 mg/dL (95% CI: −11.78 to −5.14), respectively. These results indicate that AM is effective in lowering LDL-C, with the greatest effect seen in China at the lower dose, though the magnitude of reduction varied by country and dose.

Based on the subgroup analysis of AM’s effect on LDL-C stratified by dosage and treatment duration, the results showed notable differences between groups, as shown in Supplementary Figure S6A,B. For AM < 50 mg/kg/day, the pooled mean difference was −29.76 mg/dL (95% CI: −52.06 to −7.47, *p* = 0.01), indicating a significant reduction in LDL-C. This subgroup included studies with treatment durations of <8 weeks and showed high heterogeneity (*I*^2^ = 99.92%), suggesting substantial variability among studies. For AM ≥ 50 mg/kg/day, which also included studies with treatment durations of <8 weeks, the pooled mean difference was −11.67 mg/dL (95% CI: −21.15 to −2.20, *p* = 0.02), also indicating a significant LDL-C-lowering effect, though to a lesser extent. The heterogeneity in this subgroup was moderate (*I*^2^ = 56.11%). However, the subgroup analysis by duration is not essential or conclusive, as it lacks data from studies with treatment periods between 8 and 12 weeks, limiting its ability to assess the true impact of treatment duration on LDL-C outcomes.

#### 3.5.4. Subgroup Analysis of the Effect of AM on HDL-C Levels

The subgroup analysis of AM’s effect on TC by animal species revealed contrasting outcomes, as shown in Supplementary Figure S7A,B. In C57BL/6 mice, one study showed a non-significant decrease of −2.03 mg/dL (95% CI: −12.93 to 8.87). In Wistar rats, the overall effect favored AM, although individual study results varied; the pooled estimate showed a trend toward increased HDL-C with considerable heterogeneity (*I*^2^ = 99.41%). At higher doses at ≥50 mg/kg/day, in C57BL/6 mice, a significant decrease in HDL-C was observed (−15.25 mg/dL, 95% CI: −28.43 to −2.07). In contrast, Sprague-Dawley rats showed a significant increase in HDL-C (9.28 mg/dL, 95% CI: −1.14 to 19.70). AM at <50 mg/kg/day appears to increase HDL-C, especially in Wistar rat models, while at ≥50 mg/kg/day, the effects are inconsistent, with C57BL/6 mice showing reductions and Sprague-Dawley rats showing increases.

The subgroup analysis of AM’s effect on HDL-C, stratified by dosage and country (with each country represented by one study), showed varying outcomes, as shown in Supplementary Figure S8A,B. At AM < 50 mg/kg/day, AM significantly increased HDL-C levels overall, with a pooled mean difference of 5.42 mg/dL (95% CI: −1.33 to 12.17), despite considerable heterogeneity (*I*^2^ = 99.30%). In this group, the study from China reported a significant increase in HDL-C (12.92 mg/dL, 95% CI: 4.82 to 21.03), whereas studies from Iran and South Korea showed non-significant reductions. The test for subgroup differences was statistically significant (*p* = 0.02), suggesting possible variation by country. At AM ≥ 50 mg/kg/day, the overall effect on HDL-C was not significant, with a pooled mean difference of −2.64 mg/dL (95% CI: −26.67 to 21.39, *p* = 0.83) and considerable heterogeneity (*I*^2^ = 87.78%). Within this group, the study from China showed a significant increase (9.28 mg/dL), while the study from South Korea reported a significant decrease (−15.25 mg/dL). The test for subgroup differences was statistically significant (*p* < 0.001), indicating that country-specific factors may influence the HDL-C response to AM, particularly at higher doses.

The subgroup analysis of AM’s effect on high-density lipoprotein cholesterol (HDL-C) focused solely on studies with a treatment duration of less than 8 weeks, as there were no studies exceeding 8 weeks included in the analysis, as shown in Supplementary Figure S9A,B. The results showed that AM < 50 mg/kg/day significantly increased HDL-C levels, with a pooled mean difference of 5.42 mg/dL (95% CI: −1.33 to 12.17). However, the heterogeneity among studies was considerable (*I*^2^ = 99.30%), indicating substantial variability in effect estimates. Individual studies reported mixed results, with some showing increases and others slight decreases in HDL-C. In contrast, for the <8 weeks subgroup, AM ≥ 50 mg/kg/day showed no significant effect on HDL-C, with a pooled mean difference of −2.64 mg/dL (95% CI: −26.67 to 21.39, *p* = 0.83), and considerable heterogeneity was also observed (*I*^2^ = 87.78%). Given the absence of long-term data (>8 weeks) and the limited contrast within the available durations, this subgroup analysis provides minimal clinical insight, and treatment duration may not be an essential determinant of HDL-C response to AM based on current evidence.

### 3.6. Sensitivity Analysis and Publication Bias

**Figure 9:**
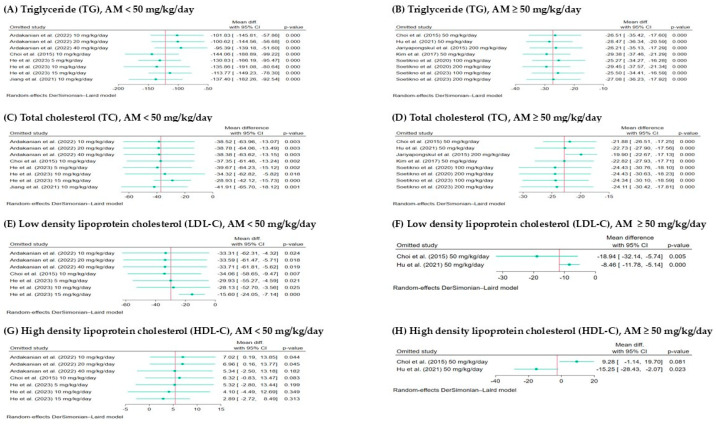
Sensitivity analysis (leave-one-out) of the effect of AM on lipid profiles stratified by dosage. Each panel shows the results of leave-one-out analysis evaluating the robustness of AM’s effect on different lipid parameters: (**A**,**B**) Triglycerides (TG), (**C**,**D**) Total cholesterol (TC), (**E**,**F**) Low-density lipoprotein cholesterol (LDL-C), (**G**,**H**) High-density lipoprotein cholesterol (HDL-C), Panels (**A**,**C**,**E**,**G**) represent AM < 50 mg/kg/day, while panels (**B**,**D**,**F**,**H**) represent AM ≥ 50 mg/kg/day. Each line displays the pooled mean difference and 95% confidence interval (CI) after omitting one study at a time. Results indicate whether any single study had a disproportionate influence on the overall estimate. The green circles represent the mean differences from omitted studies with 95% confidence intervals, and the red vertical lines indicate the overall pooled effect estimate. Analyses were conducted using a random-effects model [13,15,17,28,29,30,31,32,33].

The sensitivity analysis (leave-one-out) confirmed the robustness of AM’s triglyceride-lowering effect across both dosage groups, as shown in Figure 9A,B. For AM < 50 mg/kg/day, all analyses consistently showed significant TG reductions (mean difference range: −95.39 to −144.06 mg/dL, *p* < 0.0001), indicating no single study disproportionately influenced the results. Similarly, for AM ≥ 50 mg/kg/day, the effect remained stable (mean difference range: −25.27 to −29.45 mg/dL, *p* < 0.0001) across all iterations. These findings support the reliability of AM’s TG-lowering effect at both dose levels.

The leave-one-out sensitivity analysis demonstrated that AM’s effect on total cholesterol (TC) levels was consistent and reliable across both dosage groups, as shown in Figure 9C,D. For AM < 50 mg/kg/day, all analyses showed consistent and significant TC reductions (mean difference: −28.93 to −41.91 mg/dL, *p* < 0.05). Similarly, for AM ≥ 50 mg/kg/day, the effect remained stable (mean difference: −19.90 to −24.43 mg/dL, *p* < 0.0001). These results indicate that no single study disproportionately influenced the findings, supporting the reliability of AM’s TC-lowering effect.

The sensitivity analysis demonstrated the robustness of AM’s effect on low-density lipoprotein cholesterol (LDL-C) levels across both dosage groups in Figure 9E,F. In the AM < 50 mg/kg/day group, the LDL-C-lowering effect remained statistically significant in all iterations, with mean differences ranging from −15.60 to −34.06 mg/dL (*p* < 0.05), indicating that no single study disproportionately influenced the overall result. Similarly, in the AM ≥ 50 mg/kg/day group, the effect remained stable and significant, with mean differences of −18.94 mg/dL (*p* = 0.005) and −8.46 mg/dL (*p* < 0.0001), depending on the study omitted. These findings confirm that AM’s LDL-C-lowering effect is consistent and reliable across studies.

The sensitivity analysis for AM’s effect on HDL-C revealed that the overall findings were less stable compared to those observed for LDL-C, TC, and TG, as shown in Figure 9G,H. In the AM < 50 mg/kg/day group, the HDL-C-increasing effect remained positive in all iterations, with mean differences ranging from 2.89 to 7.02 mg/dL, but not all were statistically significant (*p* values ranging from 0.044 to 0.349), indicating potential variability in the robustness of the effect. In the AM ≥ 50 mg/kg/day group, the results were more divergent: omitting Hu et al. 2021 led to a significant increase in HDL-C (9.28 mg/dL, *p* = 0.081), while omitting Choi et al. 2015 resulted in a significant decrease (−15.25 mg/dL, *p* = 0.023) [13,17]. These findings suggest that the effect of AM on HDL-C may be less consistent and more sensitive to individual study influence, particularly at higher doses.

Due to the inclusion of only nine studies in the present meta-analysis, formal assessment of publication bias using funnel plots or statistical tests such as Egger’s test was not performed. These methods are generally considered unreliable when fewer than 10 studies are available, as the statistical power is insufficient to detect asymmetry or small-study effects.

## 4. Discussion

This systematic review and meta-analysis highlight the promising lipid-lowering effects of AM in animal models of hyperlipidemia. The pooled data from nine preclinical studies indicate that AM significantly reduces TG, TC, and LDL-C, while increasing HDL-C. These beneficial effects were observed at both low (<50 mg/kg/day) and high (≥50 mg/kg/day) dosages, although a greater magnitude of lipid reduction was generally observed with lower doses. This suggests a possible dose–response relationship, although the heterogeneity in findings—especially at lower doses—indicates other factors such as study region and experimental duration. The risk-of-bias assessment using SYRCLE’s tool indicated that most included studies had a low-to-moderate risk of bias. While outcome reporting and attrition bias were generally low, several studies lacked clear descriptions of randomization, allocation concealment, and blinding, leading to unclear risks in selection and performance domains. Despite these limitations, the overall methodological quality was acceptable, with no study showing critical bias that could significantly affect the meta-analysis results.

The results also showed that AM effectively reduced serum triglyceride (TG) levels, with the most pronounced effects observed at doses < 50 mg/kg/day, although these findings exhibited considerable heterogeneity. This heterogeneity may be attributed to differences in animal species, study region, and treatment duration. Mechanistically, AM inhibits de novo lipogenesis by downregulating SREBP-1c and FAS, while also enhancing fatty acid oxidation via PPARγ and AMPK activation [10,13]. These mechanisms reduce hepatic TG synthesis and improve peripheral TG clearance. Furthermore, AM has been reported to reduce inflammation and oxidative stress, both of which play critical roles in the pathogenesis of hypertriglyceridemia and metabolic syndrome [6]. Consistent TG-lowering effects were observed in Wistar and Sprague-Dawley rats, supporting the biological plausibility of AM’s role in lipid metabolism regulation. Nonetheless, further research is needed to explore long-term efficacy and confirm optimal dosing strategies, particularly in human subjects.

Our meta-analysis revealed that AM significantly reduced TC levels across both low (<50 mg/kg/day) and high (≥50 mg/kg/day) dosage groups. Interestingly, a greater magnitude of TC reduction was observed in studies using lower doses of AM, although with high heterogeneity. Mechanistically, AM has been shown to inhibit cholesterol biosynthesis pathways via downregulation of sterol regulatory element-binding proteins (SREBPs), particularly SREBP-1c, which modulates lipid biosynthetic genes such as fatty acid synthase (FAS) and HMG-CoA reductase [34]. Additionally, AM enhances cholesterol clearance through upregulation of AMPK and SIRT1 pathways [13,35]. These effects are consistent with other natural compounds with lipid-lowering effects, such as berberine and curcumin, which also activate AMPK-related mechanisms [36]. The variability in TC-lowering efficacy across countries and species in our subgroup analysis highlights the need for standardization in preclinical models and suggests that host metabolic context may influence the lipid-lowering efficacy of AM.

LDL-C, a major contributor to atherogenesis, was significantly reduced by AM across both dosage groups, with a greater mean reduction observed in the <50 mg/kg/day subgroup. This finding is particularly noteworthy given that even modest reductions in LDL-C are associated with significant cardiovascular benefits. The mechanism behind this effect is likely multifactorial, involving decreased hepatic lipoprotein synthesis, increased LDL receptor expression, and modulation of oxidative stress, which can impair LDL clearance [17,29]. Moreover, AM’s antioxidant properties may prevent LDL oxidation—a key event in the formation of foam cells and atherosclerotic plaques [10]. Although the studies consistently showed a lowering effect, heterogeneity remained high, especially at lower doses, suggesting possible variability in AM’s pharmacokinetics or bioavailability across species [12]. Notably, most studies included in this subgroup were of relatively short duration (<8 weeks), limiting conclusions about sustained LDL-C lowering over time.

In contrast to the effects on TC, TG, and LDL-C, lower doses of AM may be more effective in promoting HDL-C elevation, although the strong heterogeneity limits the reliability of this conclusion. AM’s ability to increase HDL-C may involve its regulatory effect on apolipoprotein A1 (ApoA1) expression, cholesterol efflux via ATP-binding cassette transporter A1 (ABCA1), and inhibition of inflammatory pathways that impair HDL maturation [37]. However, these mechanisms have not been uniformly observed across all animal models. Interestingly, in our subgroup analysis, Sprague-Dawley rats demonstrated a significant HDL-C increase at higher doses, while C57BL/6 mice showed a paradoxical decrease. This inconsistency may be attributed to interspecies differences in lipid metabolism and receptor expression. Furthermore, studies reported short intervention periods, which may be insufficient for significant HDL remodeling. The sensitivity analysis also indicated that HDL-C outcomes were more sensitive to the influence of individual studies, highlighting the need for cautious interpretation and further robust investigation.

Inflammatory regulation constitutes another major pathway through which AM confers metabolic protection. Chronic low-grade inflammation, characterized by elevated cytokines like IL-6 and TNF-α, is a hallmark of hyperlipidemia and insulin resistance. AM has been shown to suppress these pro-inflammatory cytokines by inhibiting NF-κB activation and downregulating TLR4 expression in macrophages and adipocytes [16,17]. Interestingly, AM’s antioxidant effects, mediated through increased superoxide dismutase (SOD) and glutathione (GSH) activity, also contribute to its lipid-lowering action. Oxidative stress disrupts lipid metabolism and accelerates LDL oxidation, promoting atherosclerosis. By restoring redox balance, AM may enhance endothelial function and protect against lipid peroxidation [28]. AM has been shown to reduce ceramide accumulation by inhibiting acid sphingomyelinase (aSMase), a key enzyme involved in ceramide production. Since ceramides contribute to insulin resistance and disrupted lipid metabolism, inhibiting this pathway can lead to improved lipid profiles and enhanced endothelial function [29]. These molecular changes directly contribute to reducing lipid synthesis and improving plasma lipid levels.

The subgroup analyses in this review highlight the influence of species differences, treatment duration, and country of origin on the observed effects of AM. Notably, Wistar rats and Sprague-Dawley rats consistently showed more favorable lipid profile changes, while variations across countries suggest possible influences of experimental protocols or genetic backgrounds. Interestingly, while both low and high doses of AM were effective, shorter treatment durations (<8 weeks) appeared to yield stronger effects at lower doses, whereas longer durations (8–12 weeks) enhanced the impact of higher doses, particularly on TG and TC levels. This temporal response points toward the need for optimizing treatment length and dosage in future studies.

To evaluate the robustness of the meta-analysis findings, a leave-one-out sensitivity analysis was conducted. This method systematically removes one study at a time from the pooled analysis to determine whether any single study disproportionately influences the overall effect size. In the current analysis, the leave-one-out approach revealed consistent results across all lipid parameters (TC, TG, LDL-C, and HDL-C), suggesting that no individual study significantly skewed the aggregated outcomes. This stability supports the reliability and internal validity of the meta-analysis, even in the presence of some heterogeneity. Importantly, the leave-one-out results reinforce the conclusion that AM exerts statistically robust lipid-lowering effects across diverse experimental models and conditions.

This study has several limitations. First, all included studies were conducted in animal models, limiting the generalizability of the findings to human populations. Second, there was notable heterogeneity in study designs, including differences in animal species, doses, treatment durations, and hyperlipidemia induction methods, which may have influenced the pooled outcomes. Third, several studies lacked adequate reporting on randomization and blinding, increasing the risk of bias. Additionally, the small sample sizes and limited number of studies per subgroup reduced the power of subgroup and meta-regression analyses. Finally, publication bias could not be ruled out due to the limited number of available studies and the potential for unpublished negative results. Hence, well-designed clinical trials are urgently needed to confirm these effects in humans and to determine optimal therapeutic strategies.

In summary, this meta-analysis provides strong preclinical evidence supporting the lipid-lowering effects of AM in various animal models of hyperlipidemia. The compound significantly reduced TG, TC, and LDL-C levels while increasing HDL-C, with low doses (<50 mg/kg/day) often showing greater efficacy than higher doses. These effects are likely mediated through modulation of lipid metabolism, anti-inflammatory pathways, and oxidative stress reduction. The findings were consistent across sensitivity analyses and remained robust despite moderate heterogeneity and methodological limitations. Collectively, these results highlight the therapeutic potential of AM as a natural compound for dyslipidemia management and underscore the need for well-designed clinical trials to validate its efficacy and safety in humans.

## 5. Conclusions

This systematic review and meta-analysis provide robust preclinical evidence that AM exerts significant lipid-lowering effects in animal models of hyperlipidemia. AM consistently reduced serum TG, TC, and LDL-C, while increasing HDL-C. These effects were more pronounced at lower doses (<50 mg/kg/day), suggesting a dose-dependent relationship, though heterogeneity across studies indicates that factors such as species, study region, and treatment duration also play a role. The underlying mechanisms are likely multifactorial, involving the downregulation of lipogenic genes, enhancement of fatty acid oxidation, reduction in inflammation, and mitigation of oxidative stress. Although the overall risk of bias was low-to-moderate and sensitivity analyses confirmed the robustness of the results, the findings remain limited by the exclusive use of animal models and variable study quality. Future research, particularly well-designed clinical trials, is necessary to confirm these findings in humans and to optimize dosing regimens for potential therapeutic application in dyslipidemia and metabolic syndrome.

## Figures and Tables

**Table 1:** PICO (participants, intervention/exposure, comparison, outcomes, and study design) criteria for the inclusion and exclusion of studies on the effects of alpha-mangostin on lipid profiles.

Parameter	Description
Population (P)	Animal models with induced hyperlipidemia or naturally occurring elevated lipid profiles.
Intervention (I)	Administration of alpha-mangostin in varying dosages and routes.
Comparator (C)	Vehicle-treated or placebo-treated control groups.
Outcome (O)	Changes in lipid profiles, including triglycerides (TG), total cholesterol (TC), low-density lipoprotein cholesterol (LDL-C), and high-density lipoprotein cholesterol (HDL-C).
Study design (S)	Experimental animal studies with control groups.

**Table 2:** Detailed characteristics of studies evaluating the lipid-lowering effects of alpha-mangostin included in the systematic review and meta-analysis.

Study ID	Year	Study Region	Type of Alpha-Mangostin	Route	Duration	Animal Species	Models	Number (Treatment/Control)	Treatment	Outcomes (Lipid Profile)
Ardakanian et al. [32]	2022	Iran	Purified alpha-mangostin (>90%)	Intraperitoneal injection	14 days	Wistar rats	Olanzapine-induced metabolic disorders	(6/6)	10, 20, 40 mg/kg/day	TC, TG, HDL-C, LDL-C
Choi et al. [13]	2015	South Korea	Purified alpha-mangostin (>98%)	Oral	7 days	C57BL/6 mice	High-fat diet-induced hepatic steatosis and obesity	(8/8)	10, 50 mg/kg/day	TC, TG, HDL-C, LDL-C
He et al. [28]	2023	China	Purified alpha-mangostin	Oral	18 days	Swiss albino Wistar rats	Streptozotocin-induced gestational diabetes	(8/8)	5, 10, 15 mg/kg/day	TC, TG, HDL-C, LDL-C
Hu et al. [17]	2021	China	Purified alpha-mangostin	Oral	30 days	Sprague-Dawley rats	Adjuvant-Induced Arthritis	(6/6)	50 mg/kg/day	TC, TG, HDL-C, LDL-C
Jariyapongskul et al. [33]	2015	Thailand	Purified alpha-mangostin (>95%)	Oral	8 weeks	Sprague-Dawley rats	Streptozotocin-induced Type 2 diabeted mellitus	(7/7)	200 mg/kg/day	TC, TG
Jiang et al. [29]	2021	China	Purified alpha-mangostin (≥98%)	Intraperitoneal injection	12 weeks	C57BL/KsJ-diabetic (db/db) mice	Diabetes mellitus	(8/8)	10 mg/kg/day	TC, TG
Kim et al. [30]	2017	South Korea	Purified alpha-mangostin (95–99%)	Oral	12 weeks	C57BL/6 mice	High-fat diet-induced obesity	(10/10)	50 mg/kg/day	TC, TG
Soetikno et al. [31]	2020	Indonesia	Purified alpha-mangostin (>98%)	Oral	8 weeks	Wistar rats	High-fat/high-glucose diet and streptozotocin-induced Type 2 diabetes mellitus	(6/6)	100, 200 mg/kg/day	TC, TG
Soetikno et al. [15]	2023	Indonesia	Purified alpha-mangostin (≥98%)	Oral	11 weeks	Sprague-Dawley rats	High-fat/high-glucose diet and streptozotocin-induced insulin resistance	(6/6)	100, 200 mg/kg/day	TC, TG

TG, triglyceride; TC, total cholesterol; LDL-C, low-density lipoprotein cholesterol; HDL-C, high-density lipoprotein cholesterol.

## Data Availability

No new data were created or analyzed in this study. Data sharing is not applicable to this article.

## Supplementary Materials

The following supporting information can be downloaded at: https://www.mdpi.com/article/10.3390/foods14111880/s1, Table S1: PRISMA 2020 Checklist; Table S2: Literature search in PubMed; Table S3. Literature search in Scopus; Table S4. Literature search in Embase; Table S5. Literature search in Web of Science; Supplementary Table S6. Literature search in Cochrane Library. Figure S1: Subgroup analysis of the effect of alpha-mangostin (AM) on total cholesterol (TC) levels stratified by dosage and species: (A) AM < 50 mg/kg/day: subgrouped by species (C57BL/6 mice, C7BL/KsJ-diabetic (db/db) mice and Wistar rats); (B) AM ≥ 50 mg/kg/day: subgrouped by species (C57BL/6 mice, Sprague-Dawley rats, and Wistar rats). Each subgroup presents the mean difference in TC levels (mg/dL) between the AM-treated and control groups with corresponding 95% confidence intervals (CIs). The analysis was conducted using a random-effects model. Figure S2. Subgroup analysis of the effect of alpha-mangostin (AM) on total cholesterol (TC) levels by country: (A) AM < 50 mg/kg/day: studies conducted in China, Iran, and South Korea. (B) AM ≥ 50 mg/kg/day: studies conducted in China, Indonesia, South Korea, and Thailand. Each subgroup presents the mean difference in TC levels (mg/dL) between AM-treated and control groups with corresponding 95% confidence intervals (CIs). Analysis was performed using a random-effects model to account for between-study heterogeneity. Figure S3. Subgroup analysis of the effect of AM on total cholesterol (TC) levels stratified by treatment duration: (A) Studies using AM < 50 mg/kg/day categorized by treatment durations of 8–12 weeks and <8 weeks. (B) Studies using AM ≥ 50 mg/kg/day categorized by treatment durations of 8–12 weeks and <8 weeks. Each forest plot displays the mean difference in TC levels (mg/dL) between AM-treated and control groups with 95% confidence intervals (CIs). Subgroup heterogeneity and overall effects were assessed using a random-effects model. Figure S4. Subgroup analysis of the effect of alpha-mangostin (AM) on low-density lipoprotein cholesterol (LDL-C) levels stratified by species: (A) AM < 50 mg/kg/day: subgrouped by species (C57BL/6 mice and Wistar rats); (B) AM ≥ 50 mg/kg/day: subgrouped by species (C57BL/6 mice and Sprague-Dawley rats). Each subgroup presents the mean difference in LDL-C levels (mg/dL) between the AM-treated and control groups with corresponding 95% confidence intervals (CIs). The analysis was conducted using a random-effects model. Figure S5. Subgroup analysis of the effect of alpha-mangostin (AM) on low-density lipoprotein cholesterol (LDL-C) levels by country: (A) AM < 50 mg/kg/day: studies conducted in China, Iran, and South Korea. (B) AM ≥ 50 mg/kg/day: studies conducted in China and South Korea. Each subgroup presents the mean difference in LDL-C levels (mg/dL) between AM-treated and control groups with corresponding 95% confidence intervals (CIs). Analysis was performed using a random-effects model to account for between-study heterogeneity. Figure S6. Subgroup analysis of the effect of AM on low-density lipoprotein cholesterol (LDL-C) levels stratified by treatment duration: (A) Studies using AM < 50 mg/kg/day with treatment durations of <8 weeks. (B) Studies using AM ≥ 50 mg/kg/day with treatment durations of <8 weeks. Each forest plot displays the mean difference in LDL-C levels (mg/dL) between AM-treated and control groups with 95% confidence intervals (CIs). Subgroup heterogeneity and overall effects were assessed using a random-effects model. Figure S7. Subgroup analysis of the effect of alpha-mangostin (AM) on high-density lipoprotein cholesterol (HDL-C) levels stratified by species: (A) AM < 50 mg/kg/day: subgrouped by species (C57BL/6 mice and Wistar rats); (B) AM ≥ 50 mg/kg/day: subgrouped by species (C57BL/6 mice and Sprague-Dawley rats). Each subgroup presents the mean difference in TG levels (mg/dL) between the AM-treated and control groups with corresponding 95% confidence intervals (CIs). The analysis was conducted using a random-effects model. Figure S8. Subgroup analysis of the effect of alpha-mangostin (AM) on high-density lipoprotein cholesterol (HDL-C) levels by country: (A) AM < 50 mg/kg/day: studies conducted in China, Iran, and South Korea. (B) AM ≥ 50 mg/kg/day: studies conducted in China and South Korea. Each subgroup presents the mean difference in HDL-C levels (mg/dL) between AM-treated and control groups with corresponding 95% confidence intervals (CIs). Analysis was performed using a random-effects model to account for between-study heterogeneity. Figure S9. Subgroup analysis of the effect of AM on high-density lipoprotein cholesterol (HDL-C) levels stratified by treatment duration: (A) Studies using AM < 50 mg/kg/day with treatment durations of <8 weeks. (B) Studies using AM ≥ 50 mg/kg/day with treatment durations of <8 weeks. Each forest plot displays the mean difference in HDL-C levels (mg/dL) between AM-treated and control groups with 95% confidence intervals (CIs). Subgroup heterogeneity and overall effects were assessed using a random-effects model.
