# Supplementary material for: Lipid-Lowering Effects of Alpha-Mangostin: A Systematic Review and Meta-Analysis in Hyperlipidemic Animal Models
Source: Foods. 2025 May 26;14(11):1880. doi: 10.3390/foods14111880 (PMC12154098; doi:10.3390/foods14111880)
Supplement: Supplementary file 1 [file foods-14-01880-s001.zip › foods-3624351-supplementary.pdf]

Lipid-Lowering Effects of Alpha-Mangostin: A Systematic Review and Meta-Analysis in Hyperlipidemic Animal Models

Moragot Chatatikun [1,2], Aman Tedasen [1,2], Phichayut Phinyo [3,4], Pakpoom Wongyikul [3,4], Passakorn Poolbua [1], Wiyada Kwanhian Klangbud [5], Jason C. Huang [6], Rattana Leelawattana [7] and Atthaphong Phongphithakchai [8,*]

1 Department of Medical Technology, School of Allied Health Sciences, Walailak University, Nakhon Si Thammarat 80160, Thailand; moragot.ch@wu.ac.th (M.C.); aman.te@wu.ac.th (A.T.); patsakorn.po@mail.wu.ac.th (P.P.)
2 Research Excellence Center for Innovation and Health Products (RECIHP), Walailak University, Nakhon Si Thammarat 80160, Thailand
3 Center for Clinical Epidemiology and Clinical Statistics, Faculty of Medicine, Chiang Mai University, Chiang Mai 50200, Thailand; phichayut.phinyo@cmu.ac.th (P.P.); aumkidify@gmail.com (P.W.)
4 Department of Biomedical Informatics and Clinical Epidemiology (BioCE), Faculty of Medicine, Chiang Mai University, Chiang Mai 50200, Thailand
5 Medical Technology Program, Faculty of Science, Nakhon Phanom University, Nakhon Phanom 48000, Thailand; wiyadakwanhian@gmail.com
6 Department of Biotechnology and Laboratory Science in Medicine, National Yang Ming Chiao Tung University, Taipei 112304, Taiwan; jasonhuang@nycu.edu.tw
7 Endocrinology and Metabolism Unit, Division of Internal Medicine, Faculty of Medicine, Prince of Songkla University, Songkhla 90110, Thailand; lrattana@meicine.psu.ac.th
8 Nephrology Unit, Division of Internal Medicine, Faculty of Medicine, Prince of Songkla University, Songkhla 90110, Thailand
* Correspondence: atthaphong.p@psu.ac.th

Supplementary Table S1. PRISMA 2020 Checklist

Section and Topic	Item #	Checklist item	Location where item is reported
**TITLE**			
Title	1	Identify the report as a systematic review.	Page 1
**ABSTRACT**			
Abstract	2	See the PRISMA 2020 for Abstracts checklist.	Page 1
**INTRODUCTION**			
Rationale	3	Describe the rationale for the review in the context of existing knowledge.	Page 2
Objectives	4	Provide an explicit statement of the objective(s) or question(s) the review addresses.	Page 2
**METHODS**			
Eligibility criteria	5	Specify the inclusion and exclusion criteria for the review and how studies were grouped for the syntheses.	Page 3
Information sources	6	Specify all databases, registers, websites, organisations, reference lists and other sources searched or consulted to identify studies. Specify the date when each source was last searched or consulted.	Page 3
Search strategy	7	Present the full search strategies for all databases, registers and websites, including any filters and limits used.	Supplementary Tables S2–S6
Selection process	8	Specify the methods used to decide whether a study met the inclusion criteria of the review, including how many reviewers screened each record and each report retrieved, whether they worked independently, and if applicable, details of automation tools used in the process.	Page 3
Data collection process	9	Specify the methods used to collect data from reports, including how many reviewers collected data from each report, whether they worked independently, any processes for obtaining or confirming data from study investigators, and if applicable, details of automation tools used in the process.	Page 4
Data items	10a	List and define all outcomes for which data were sought. Specify whether all results that were compatible with each outcome domain in each study were sought (e.g. for all measures, time points, analyses), and if not, the methods used to decide which results to collect.	Page 4
	10b	List and define all other variables for which data were sought (e.g. participant and intervention characteristics, funding sources). Describe any assumptions made about any missing or unclear information.	Page 4
Study risk of bias assessment	11	Specify the methods used to assess risk of bias in the included studies, including details of the tool(s) used, how many reviewers assessed each study and whether they worked independently, and if applicable, details of automation tools used in the process.	Pages 4
Effect measures	12	Specify for each outcome the effect measure(s) (e.g. risk ratio, mean difference) used in the synthesis or presentation of results.	Page 4
Synthesis methods	13a	Describe the processes used to decide which studies were eligible for each synthesis (e.g. tabulating the study intervention characteristics and comparing against the planned groups for each synthesis (item #5)).	Pages 4-5
	13b	Describe any methods required to prepare the data for presentation or synthesis, such as handling of missing summary statistics, or data conversions.	Pages 4-5

Supplementary Table S1. PRISMA 2020 Checklist

Section and Topic	Item #	Checklist item	Location where item is reported
	13c	Describe any methods used to tabulate or visually display results of individual studies and syntheses.	Pages 4-5
	13d	Describe any methods used to synthesize results and provide a rationale for the choice(s). If meta-analysis was performed, describe the model(s), method(s) to identify the presence and extent of statistical heterogeneity, and software package(s) used.	Pages 4-5
	13e	Describe any methods used to explore possible causes of heterogeneity among study results (e.g. subgroup analysis, meta-regression).	Pages 4-5
	13f	Describe any sensitivity analyses conducted to assess robustness of the synthesized results.	Pages 4-5
Reporting bias assessment	14	Describe any methods used to assess risk of bias due to missing results in a synthesis (arising from reporting biases).	Pages 4
Certainty assessment	15	Describe any methods used to assess certainty (or confidence) in the body of evidence for an outcome.	Pages 4-5
**RESULTS**			
Study selection	16a	Describe the results of the search and selection process, from the number of records identified in the search to the number of studies included in the review, ideally using a flow diagram.	Page 5
	16b	Cite studies that might appear to meet the inclusion criteria, but which were excluded, and explain why they were excluded.	Page 5
Study characteristics	17	Cite each included study and present its characteristics.	Page 6-8
Risk of bias in studies	18	Present assessments of risk of bias for each included study.	Page 9
Results of individual studies	19	For all outcomes, present, for each study: (a) summary statistics for each group (where appropriate) and (b) an effect estimate and its precision (e.g. confidence/credible interval), ideally using structured tables or plots.	Pages 9-18
Results of syntheses	20a	For each synthesis, briefly summarise the characteristics and risk of bias among contributing studies.	Pages 5-9
	20b	Present results of all statistical syntheses conducted. If meta-analysis was done, present for each the summary estimate and its precision (e.g. confidence/credible interval) and measures of statistical heterogeneity. If comparing groups, describe the direction of the effect.	Pages 9-18
	20c	Present results of all investigations of possible causes of heterogeneity among study results.	Pages 9-16
	20d	Present results of all sensitivity analyses conducted to assess the robustness of the synthesized results.	Pages 16-18
Reporting biases	21	Present assessments of risk of bias due to missing results (arising from reporting biases) for each synthesis assessed.	Page 9
Certainty of evidence	22	Present assessments of certainty (or confidence) in the body of evidence for each outcome assessed.	Page 9
**DISCUSSION**			

Supplementary Table S1. PRISMA 2020 Checklist

Section and Topic	Item #	Checklist item	Location where item is reported
Discussion	23a	Provide a general interpretation of the results in the context of other evidence.	Pages 18–20
	23b	Discuss any limitations of the evidence included in the review.	Page 18–20
	23c	Discuss any limitations of the review processes used.	Page 18-20
	23d	Discuss implications of the results for practice, policy, and future research.	Page 20
**OTHER INFORMATION**			
Registration and protocol	24a	Provide registration information for the review, including register name and registration number, or state that the review was not registered.	Page 2
	24b	Indicate where the review protocol can be accessed, or state that a protocol was not prepared.	Page 2
	24c	Describe and explain any amendments to information provided at registration or in the protocol.	Not applicable
Support	25	Describe sources of financial or non-financial support for the review, and the role of the funders or sponsors in the review.	Page 22
Competing interests	26	Declare any competing interests of review authors.	
Availability of data, code and other materials	27	Report which of the following are publicly available and where they can be found: template data collection forms; data extracted from included studies; data used for all analyses; analytic code; any other materials used in the review.	Page 22

Supplementary Table S2. Literature search in PubMed.

Search Number	Query	Search Details	Results
**1**	Alpha-mangostin[Title/Abstract]	"Alpha-mangostin"[Title/Abstract]	629
**2**	mangostin[Title/Abstract]	"mangostin"[Title/Abstract]	741
**3**	Garcinia mangostana[Title/Abstract]	"garcinia mangostana"[Title/Abstract]	559
**4**	Garcinia mangostana[MeSH Terms]	"garcinia mangostana"[MeSH Terms]	369
**5**	mangosteen[Title/Abstract]	"mangosteen"[Title/Abstract]	559
**6**	mangosteen[MeSH Terms]	"garcinia mangostana"[MeSH Terms]	369
**7**	#1 OR #2 OR #3 OR #4 OR #5 OR #6	"Alpha-mangostin"[Title/Abstract] OR "mangostin"[Title/Abstract] OR "garcinia mangostana"[Title/Abstract] OR "garcinia mangostana"[MeSH Terms] OR "mangosteen"[Title/Abstract] OR "garcinia mangostana"[MeSH Terms]	1,193
**8**	Lipid profile[Title/Abstract]	"lipid profile"[Title/Abstract]	30,856
**9**	Cholesterol[Title/Abstract]	"Cholesterol"[Title/Abstract]	291,848
**10**	cholesterol[MeSH Terms]	"cholesterol"[MeSH Terms]	177,664
**11**	Triglyceride[Title/Abstract]	"Triglyceride"[Title/Abstract]	75,748
**12**	triglyceride[MeSH Terms]	"triglycerides"[MeSH Terms]	86,755
**13**	low-density lipoprotein[Title/Abstract]	"low density lipoprotein"[Title/Abstract]	83,834
**14**	low-density lipoprotein[MeSH Terms]	"lipoproteins, ldl"[MeSH Terms]	61,548
**15**	LDL[Title/Abstract]	"LDL"[Title/Abstract]	93,851
**16**	high-density lipoprotein[Title/Abstract]	"high density lipoprotein"[Title/Abstract]	62,036
**17**	high-density lipoprotein[MeSH Terms]	"lipoproteins, hdl"[MeSH Terms]	48,858
**18**	HDL[Title/Abstract]	"HDL"[Title/Abstract]	80,002
**19**	Lipids[Title/Abstract]	"Lipids"[Title/Abstract]	185,584
**20**	lipids[MeSH Terms]	"lipids"[MeSH Terms]	1,314,653

**21**	#8 OR #9 OR #10 OR #11 OR #12 OR #13 OR #14 OR #15 OR #16 OR #17 OR #18 OR #19 OR #20	"lipid profile"[Title/Abstract] OR "Cholesterol"[Title/Abstract] OR "Cholesterol"[MeSH Terms] OR "Triglyceride"[Title/Abstract] OR "triglycerides"[MeSH Terms] OR "low density lipoprotein"[Title/Abstract] OR "lipoproteins, ldl"[MeSH Terms] OR "LDL"[Title/Abstract] OR "high density lipoprotein"[Title/Abstract] OR "lipoproteins, hdl"[MeSH Terms] OR "HDL"[Title/Abstract] OR "Lipids"[Title/Abstract] OR "Lipids"[MeSH Terms]	1,529,387
**22**	Animal model[Title/Abstract]	"animal model"[Title/Abstract]	97,239
**23**	Animal model[MeSH Terms]	"models, animal"[MeSH Terms]	656,360
**24**	Animal models[Title/Abstract]	"animal models"[Title/Abstract]	139,584
**25**	Animal models[MeSH Terms]	"models, animal"[MeSH Terms]	656,360
**26**	animal[Title/Abstract]	"animal"[Title/Abstract]	642,074
**27**	animal[MeSH Terms]	"animals"[MeSH Terms]	27,344,841
**28**	animals[Title/Abstract]	"animals"[Title/Abstract]	812,444
**29**	animals[MeSH Terms]	"animals"[MeSH Terms]	27,344,841
**30**	Mice[Title/Abstract]	"Mice"[Title/Abstract]	1,109,252
**31**	Mice[MeSH Terms]	"mice"[MeSH Terms]	1,851,599
**32**	Rat[Title/Abstract]	"Rat"[Title/Abstract]	926,364
**33**	rat[MeSH Terms]	"rats"[MeSH Terms]	1,721,050
**34**	rats[Title/Abstract]	"rats"[Title/Abstract]	939,150
**35**	rats[MeSH Terms]	"rats"[MeSH Terms]	1,721,050
**36**	rodent[Title/Abstract]	"rodent"[Title/Abstract]	75,246
**37**	Rodent[MeSH Terms]	"rodentia"[MeSH Terms]	3,627,317
**38**	In vivo[Title/Abstract]	"in vivo"[Title/Abstract]	1,114,391
**39**	preclinical[Title/Abstract]	"preclinical"[Title/Abstract]	165,912
**40**	#22 OR #23 OR #24 OR #25 OR #26 OR #27 OR #28 OR #29 OR #30 OR #31 OR #32 OR #33 OR #34 OR #35 OR #36 OR #37 OR #38 OR #39	"animal model"[Title/Abstract] OR "models, animal"[MeSH Terms] OR "animal models"[Title/Abstract] OR "models, animal"[MeSH Terms] OR "Animal"[Title/Abstract] OR "animals"[MeSH Terms] OR "animals"[Title/Abstract] OR "animals"[MeSH Terms] OR	27,835,865

		"Mice"[Title/Abstract] OR "Mice"[MeSH Terms] OR "Rat"[Title/Abstract] OR "rats"[MeSH Terms] OR "rats"[Title/Abstract] OR "rats"[MeSH Terms] OR "rodent"[Title/Abstract] OR "rodentia"[MeSH Terms] OR "in vivo"[Title/Abstract] OR "preclinical"[Title/Abstract]	
**41**	#7 AND #21 AND #40	("Alpha-mangostin"[Title/Abstract] OR "mangostin"[Title/Abstract] OR "garcinia mangostana"[Title/Abstract] OR "garcinia mangostana"[MeSH Terms] OR "mangosteen"[Title/Abstract] OR "garcinia mangostana"[MeSH Terms]) AND ("lipid profile"[Title/Abstract] OR "Cholesterol"[Title/Abstract] OR "Cholesterol"[MeSH Terms] OR "Triglyceride"[Title/Abstract] OR "triglycerides"[MeSH Terms] OR "low density lipoprotein"[Title/Abstract] OR "lipoproteins, ldl"[MeSH Terms] OR "LDL"[Title/Abstract] OR "high density lipoprotein"[Title/Abstract] OR "lipoproteins, hdl"[MeSH Terms] OR "HDL"[Title/Abstract] OR "Lipids"[Title/Abstract] OR "Lipids"[MeSH Terms]) AND ("animal model"[Title/Abstract] OR "models, animal"[MeSH Terms] OR "animal models"[Title/Abstract] OR "models, animal"[MeSH Terms] OR "Animal"[Title/Abstract] OR "animals"[MeSH Terms] OR "animals"[Title/Abstract] OR "animals"[MeSH Terms] OR "Mice"[Title/Abstract] OR "Mice"[MeSH Terms] OR "Rat"[Title/Abstract] OR "rats"[MeSH Terms] OR "rats"[Title/Abstract] OR "rats"[MeSH Terms] OR "rodent"[Title/Abstract] OR "rodentia"[MeSH Terms] OR "in vivo"[Title/Abstract] OR "preclinical"[Title/Abstract])	88

Supplementary Table S3. Literature search in Scopus.

Search Number	Search Details	Results
#1	TITLE-ABS-KEY (alpha-mangostin)	1,069
#2	TITLE-ABS-KEY (mangostin)	1,339
#3	TITLE-ABS-KEY (garcinia AND mangostana)	1,765
#4	TITLE-ABS-KEY (mangosteen)	1,755
#5	(TITLE-ABS-KEY (alpha-mangostin)) OR (TITLE-ABS-KEY (mangostin)) OR(TITLE-ABS-KEY (garcinia AND mangostana)) OR (TITLE-ABS-KEY (mangosteen))	3,117
#6	TITLE-ABS-KEY (lipid AND profile)	115,016
#7	TITLE-ABS-KEY (cholesterol)	536,479
#8	TITLE-ABS-KEY (triglyceride)	212,337
#9	TITLE-ABS-KEY (low-density AND lipoprotein)	243,385
#10	TITLE-ABS-KEY (LDL)	139,363
#11	TITLE-ABS-KEY (high-density AND lipoprotein)	194,269
#12	TITLE-ABS-KEY (hdl)	121,132
#13	TITLE-ABS-KEY (lipids)	110,4142
#14	(TITLE-ABS-KEY (lipid AND profile)) OR (TITLE-ABS-KEY (cholesterol)) OR (TITLE-ABS-KEY (triglyceride)) OR (TITLE-ABS-KEY (low-densityAND lipoprotein)) OR (TITLE-ABS-KEY (ldl)) OR (TITLE-ABS-KEY (high-density AND lipoprotein)) OR (TITLE-ABS-KEY (hdl)) OR (TITLE-ABS-KEY (lipids))	1,511,501
#15	ITLE-ABS-KEY (ANIMAL AND MODEL)	2,604,677
#16	TITLE-ABS-KEY (animal AND models)	2,604,677
#17	TITLE-ABS-KEY (ANIMAL)	8,501,197
#18	TITLE-ABS-KEY (ANIMALS)	8,501,197
#19	TITLE-ABS-KEY (MICE)	2,423,323
#20	TITLE-ABS-KEY (RAT)	2,258,976
#21	TITLE-ABS-KEY (RATS)	2,258,976
#22	TITLE-ABS-KEY (IN AND VIVO)	1,469,039
#23	TITLE-ABS-KEY (PRECLINICAL)	210,327
#24	(TITLE-ABS-KEY (ANIMAL AND MODEL)) OR (TITLE-ABS-KEY (ANIMAL AND MODELS)) OR (TITLE-ABS-KEY (ANIMAL)) OR (TITLE-ABS-KEY (ANIMALS)) OR (TITLE-ABS-KEY (MICE)) OR (TITLE-ABS-KEY (RAT)) OR (TITLE-ABS-KEY (RATS))	9,578,713

	OR (TITLE-ABS-KEY (IN AND VIVO)) OR (TITLE-ABS-KEY (PRECLINICAL))	
#25	((TITLE-ABS-KEY (ANIMAL AND MODEL)) OR (TITLE-ABS-KEY (ANIMAL AND MODELS)) OR (TITLE-ABS-KEY (ANIMAL)) OR (TITLE-ABS-KEY (ANIMALS)) OR (TITLE-ABS-KEY (MICE)) OR (TITLE-ABS-KEY (RAT)) OR (TITLE-ABS-KEY (RATS)) OR (TITLE-ABS-KEY (IN AND VIVO)) OR (TITLE-ABS-KEY (PRECLINICAL))) AND ((TITLE-ABS-KEY (LIPID AND PROFILE)) OR (TITLE-ABS-KEY (CHOLESTEROL)) OR (TITLE-ABS-KEY (TRIGLYCERIDE)) OR (TITLE-ABS-KEY (LOW-DENSITY AND LIPOPROTEIN)) OR (TITLE-ABS-KEY (LDL)) OR (TITLE-ABS-KEY (HIGH-DENSITY AND LIPOPROTEIN)) OR (TITLE-ABS-KEY (HDL)) OR (TITLE-ABS-KEY (LIPIDS))) AND ((TITLE-ABS-KEY (ALPHA-MANGOSTIN)) OR (TITLE-ABS-KEY (MANGOSTIN)) OR (TITLE-ABS-KEY (GARCINIA AND MANGOSTANA)) OR (TITLE-ABS-KEY (MANGOSTEEN)))	148

Supplementary Table S4. Literature search in Embase.

No.	Query	Results
#1	'alpha mangostin'	739
#2	'mangostin'	1,128
#3	garcinia AND mangostana	1,113
#4	'mangosteen'	785
#5	#1 OR #2 OR #3 OR #4	1,888
#6	lipid AND profile	88,217
#7	cholesterol	561,338
#8	triglyceride	106,454
#9	'low density' AND lipoprotein	283,409
#10	ldl	146,054
#11	'high density' AND lipoprotein	228,695
#12	hdl	126,894
#13	lipids	254,693
#14	#6 OR #7 OR #8 OR #9 OR #10 OR #11 OR #12 OR #13	891,182
#15	animal AND model	2,473,338
#16	animal AND models	584,303
#17	animal	7,272,524
#18	animals	1,046,504
#19	mice	1,513,267
#20	rat	2,168,398
#21	rats	1,161,345
#22	roents	0
#23	in AND vivo	1,614,974
#24	preclinical	290,005
#25	#15 OR #16 OR #17 OR #18 OR #19 OR #20 OR #21 OR #22 OR #23 OR #24	8,713,570
#26	#5 AND #14 AND #25	58

Supplementary Table S5. Literature search in Web of Science.

#	Search Query	Results
1	alpha-mangostin (All Fields)	945
2	ALL=(mangostin)	1,129
3	ALL=(garcinia mangostana)	1,285
4	ALL=(mangosteen)	1,352
5	#1 OR #2 OR #3 OR #4	2,443
6	ALL=(Lipid profile)	105,642
7	ALL=(cholesterol)	265,439
8	ALL=(triglyceride)	124,575
9	ALL=(low-density lipoprotein)	86,010
10	ALL=(ldl)	85,153
11	ALL=(high-density lipoprotein)	58,953
12	ALL=(hdl)	73,967
13	ALL=(lipids)	682,688
14	#6 OR #7 OR #8 OR #9 OR #10 OR #11 OR #12 OR #13	904,214
15	ALL=(Animal model)	559,772
16	ALL=(Animal models)	560,407
17	ALL=(animal)	1,820,106
18	ALL=(animals)	1,216,390
19	ALL=(mice)	1,470,349
20	ALL=(rat)	1,052,105
21	ALL=(rats)	1,046,465
22	ALL=(rodents)	1,223,40
23	ALL=(in vivo)	1,217,450
24	ALL=(preclinical)	150,412
25	#15 OR #16 OR #17 OR #18 OR #19 OR #20 OR #21 OR #22 OR #23 OR #24	4,452,163
26	#5 AND #14 AND #25	95

Supplementary Table S6. Literature search in Cochrane Library.

No.	Search Query	Results
#1	Alpha-mangostin	5
#2	Mangostin	12
#3	Garcinia mangostana	57
#4	mangosteen	56
#5	#1 OR #2 OR #3 OR #4<br>(mangosteen):ti,ab,kw OR (Alpha-mangostin):ti,ab,kw OR ("Garcinia mangostana"):ti,ab,kw OR (mangostin):ti,ab,kw	84
#6	Lipid profile	16,876
#7	Cholesterol	44,610
#8	Triglyceride	28,083
#9	low-density lipoprotein	17,024
#10	LDL	26,112
#11	high-density lipoprotein	13,324
#12	HDL	21,146
#13	Lipids	54,423
#14	#6 OR #7 OR #8 OR #9 OR #10 OR #11 OR #12 OR #13<br>(lipid profile):ti,ab,kw OR (cholesterol):ti,ab,kw OR (triglyeride)ti,ab,kw OR (low-density lipoprotein):ti,ab,kw OR (LDL):ti,ab,kw OR (high-density lipoprotein):ti,ab,kw OR (HDL):ti,ab,kw OR (lipids):ti,ab,kw	65,164
#15	Animal model	14,207
#16	Animal models	14,211
#17	animal	42,134
#18	animals	42,138
#19	Mice	6,332
#20	Rat	4,924
#21	Rats	4,920
#22	Rodents	1,827
#23	In vivo	20,785
#24	preclinical	11,358
#25	(animal model):ti,ab,kw OR (animal models):ti,ab,kw OR (animal):ti,ab,kw OR (animals):ti,ab,kw OR (mice):ti,ab,kw OR (rat):ti,ab,kw OR (rats):ti,ab,kw OR (rodents):ti,ab,kw OR (in vivo):ti,ab,kw OR (preclinical):ti,ab,kw	72,791
	#5 OR #14 OR #25	1

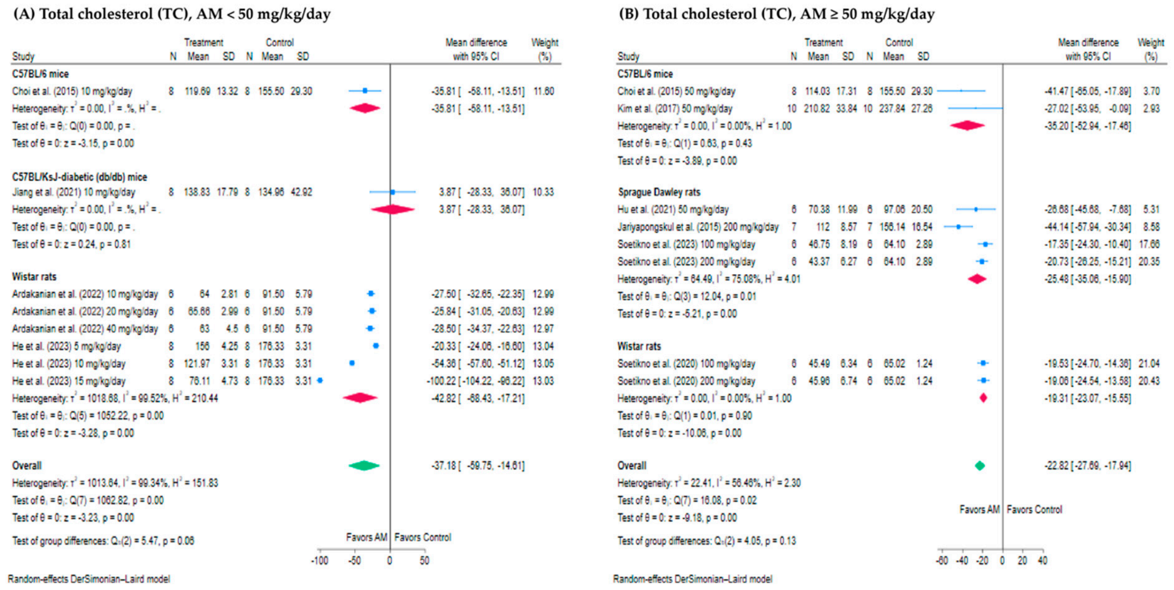

Supplementary Figure S1: Subgroup analysis of the effect of alpha-mangostin (AM) on total cholesterol (TC) levels stratified by dosage and species. (A) AM < 50 mg/kg/day: subgrouped by species (C57BL/6 mice, C7BL/KsJ-diabetic (db/db) mice and Wistar rats); (B) AM ≥ 50 mg/kg/day: subgrouped by species (C57BL/6 mice, Sprague Dawley rats, and Wistar rats). Each subgroup presents the mean difference in TC levels (mg/dL) between the AM-treated and control groups with corresponding 95% confidence intervals (CIs). Squares indicate study effect sizes with size proportional to weight; horizontal lines show 95% CIs; diamonds represent pooled effect sizes with 95% CIs. The analysis was conducted using a random-effects model [13, 15, 28–33].

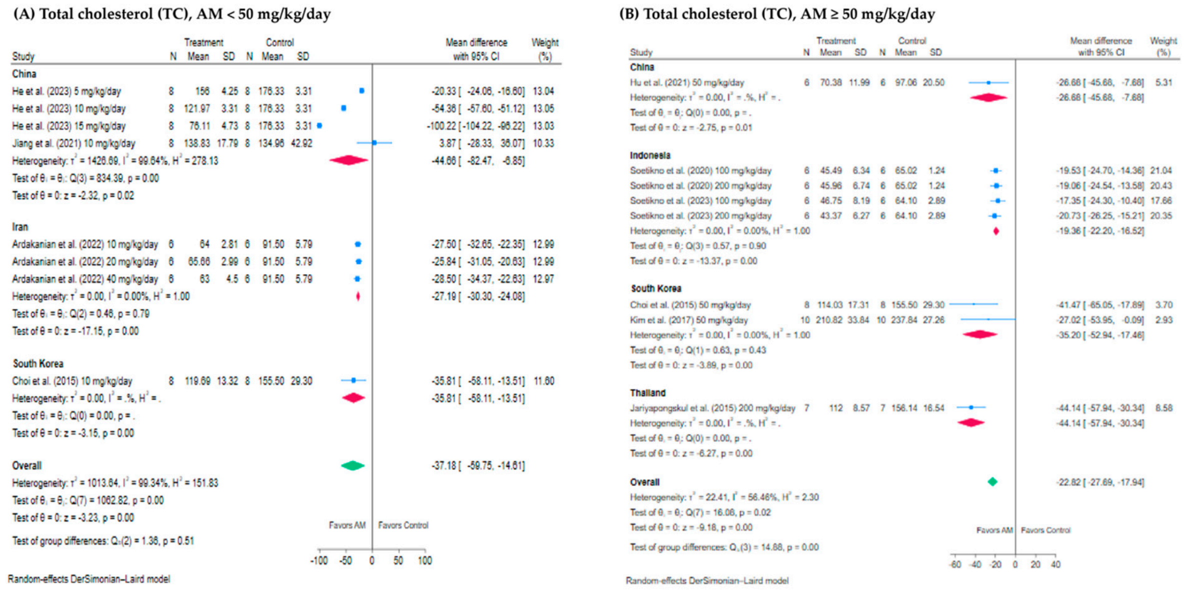

Supplementary Figure S2. Subgroup analysis of the effect of alpha-mangostin (AM) on total cholesterol (TC) levels by country. (A) AM < 50 mg/kg/day: studies conducted in China, Iran, and South Korea. (B) AM ≥ 50 mg/kg/day: studies conducted in China, Indonesia, South Korea, and Thailand. Each subgroup presents the mean difference in TC levels (mg/dL) between AM-treated and control groups with corresponding 95% confidence intervals (CIs). Squares indicate study effect sizes with size proportional to weight; horizontal lines show 95% CIs; diamonds represent pooled effect sizes with 95% CIs. Analysis was performed using a random-effects model to account for between-study heterogeneity [13, 15, 17, 28–29, 30–33].

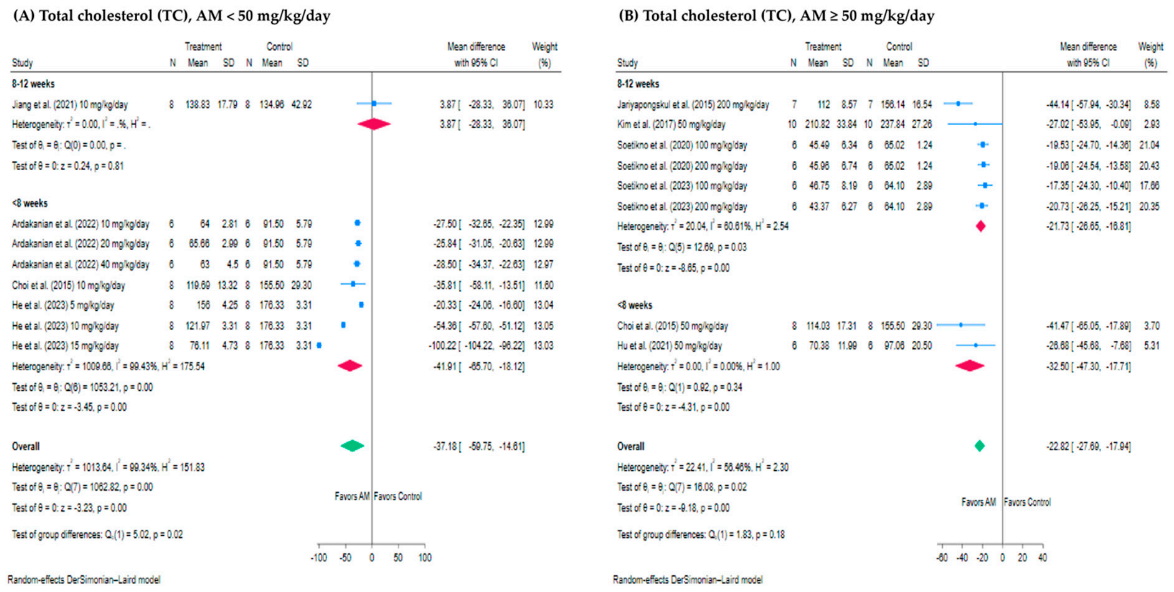

Supplementary Figure S3. Subgroup analysis of the effect of AM on total cholesterol (TC) levels stratified by treatment duration: (A) Studies using AM < 50 mg/kg/day categorized by treatment durations of 8–12 weeks and <8 weeks. (B) Studies using AM ≥ 50 mg/kg/day categorized by treatment durations of 8–12 weeks and <8 weeks. Each forest plot displays the mean difference in TC levels (mg/dL) between AM-treated and control groups with 95% confidence intervals (CIs). Squares indicate study effect sizes with size proportional to weight; horizontal lines show 95% CIs; diamonds represent pooled effect sizes with 95% CIs. Subgroup heterogeneity and overall effects were assessed using a random-effects model [13, 15, 17, 28-29, 30-33].

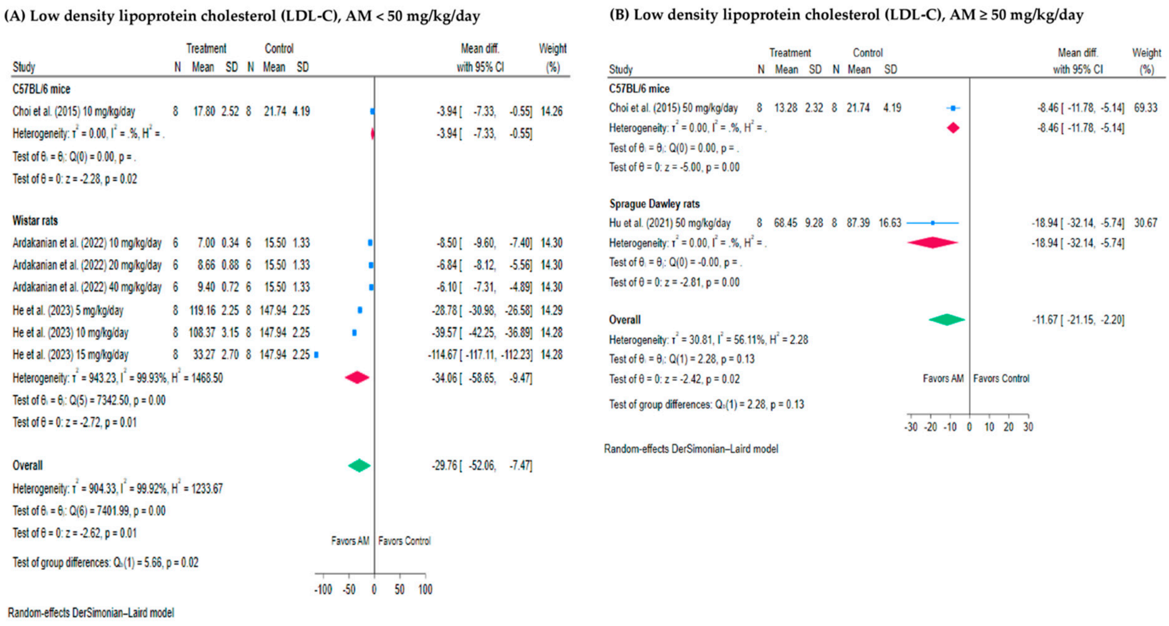

Supplementary Figure S4. Subgroup analysis of the effect of alpha-mangostin (AM) on low density lipoprotein cholesterol (LDL-C) levels stratified by species. (A) AM < 50 mg/kg/day: subgrouped by species (C57BL/6 mice and Wistar rats); (B) AM ≥ 50 mg/kg/day: subgrouped by species (C57BL/6 mice and Sprague Dawley rats). Each subgroup presents the mean difference in LDL-C levels (mg/dL) between the AM-treated and control groups with corresponding 95% confidence intervals (CIs). Squares indicate study effect sizes with size proportional to weight; horizontal lines show 95% CIs; diamonds represent pooled effect sizes with 95% CIs. The analysis was conducted using a random-effects model [13, 17, 28, 32].

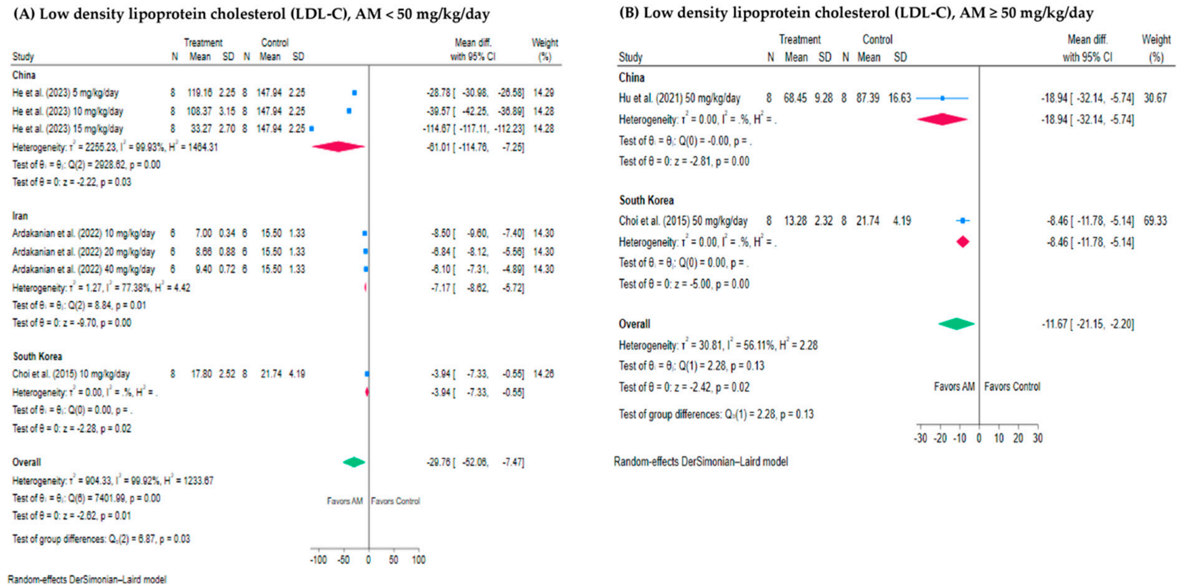

Supplementary Figure S5. Subgroup analysis of the effect of alpha-mangostin (AM) on low density lipoprotein cholesterol (LDL-C) levels by country. (A) AM < 50 mg/kg/day: studies conducted in China, Iran, and South Korea. (B) AM ≥ 50 mg/kg/day: studies conducted in China and South Korea. Each subgroup presents the mean difference in LDL-C levels (mg/dL) between AM-treated and control groups with corresponding 95% confidence intervals (CIs). Squares indicate study effect sizes with size proportional to weight; horizontal lines show 95% CIs; diamonds represent pooled effect sizes with 95% CIs. Analysis was performed using a random-effects model to account for between-study heterogeneity [13, 17, 28, 32].

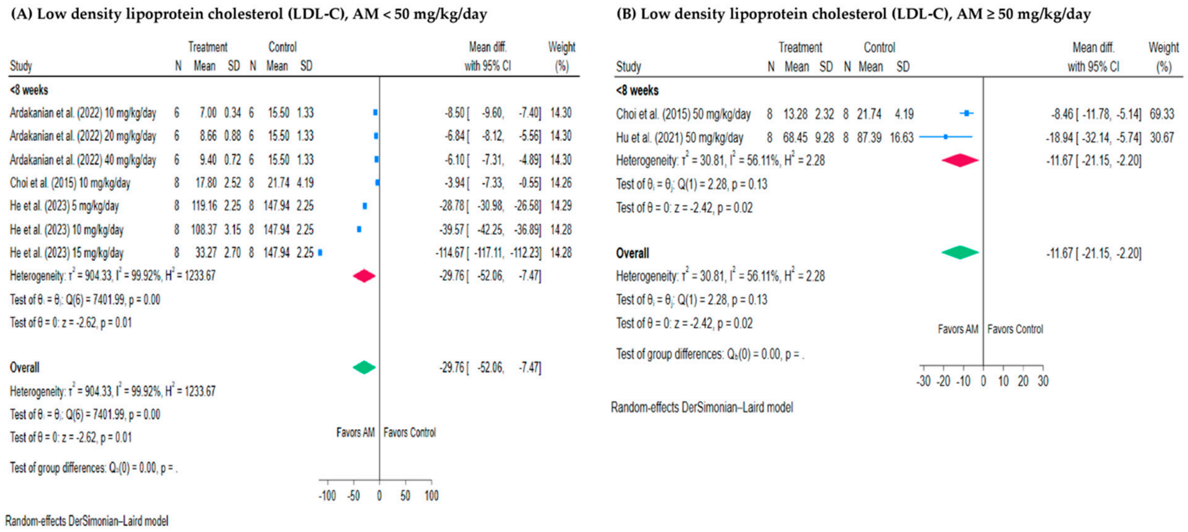

Supplementary Figure S6. Subgroup analysis of the effect of AM on low density lipoprotein cholesterol (LDL-C) levels stratified by treatment duration. (A) Studies using AM < 50 mg/kg/day with treatment durations of < 8 weeks. (B) Studies using AM ≥ 50 mg/kg/day with treatment durations of < 8 weeks. Each forest plot displays the mean difference in LDL-C levels (mg/dL) between AM-treated and control groups with 95% confidence intervals (CIs). Squares indicate study effect sizes with size proportional to weight; horizontal lines show 95% CIs; diamonds represent pooled effect sizes with 95% CIs. Subgroup heterogeneity and overall effects were assessed using a random-effects model [13, 17, 28, 32,].

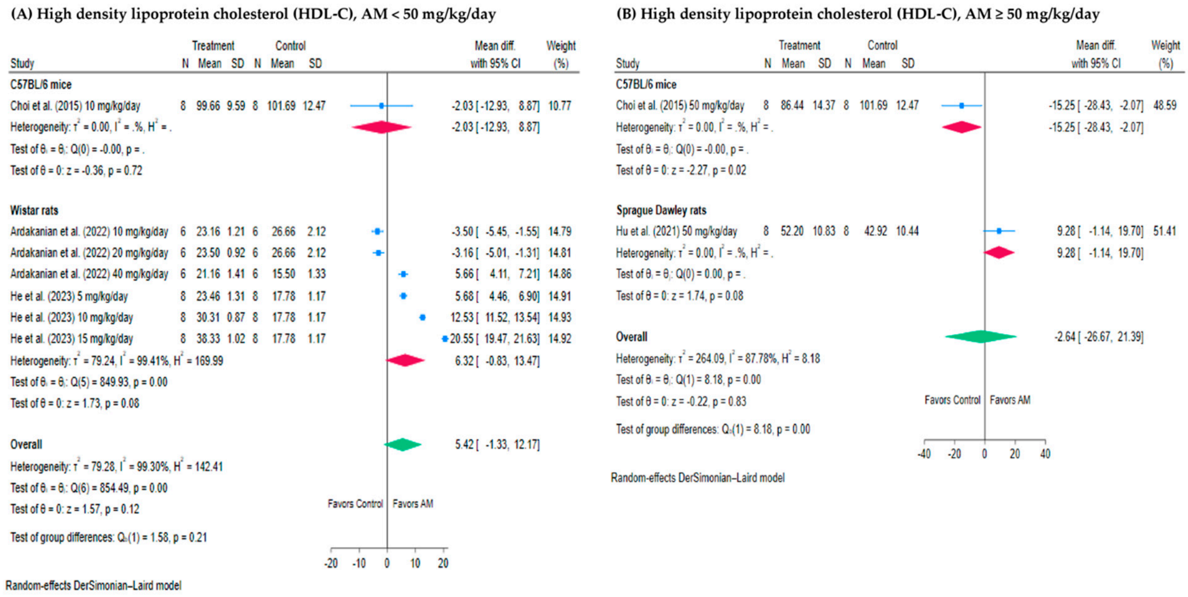

Supplementary Figure S7. Subgroup analysis of the effect of alpha-mangostin (AM) on high density lipoprotein cholesterol (HDL-C) levels stratified by species. (A) AM < 50 mg/kg/day: subgrouped by species (C57BL/6 mice and Wistar rats); (B) AM ≥ 50 mg/kg/day: subgrouped by species (C57BL/6 mice and Sprague Dawley rats). Each subgroup presents the mean difference in TG levels (mg/dL) between the AM-treated and control groups with corresponding 95% confidence intervals (CIs). Squares indicate study effect sizes with size proportional to weight; horizontal lines show 95% CIs; diamonds represent pooled effect sizes with 95% CIs. The analysis was conducted using a random-effects model [13, 17, 28, 32].

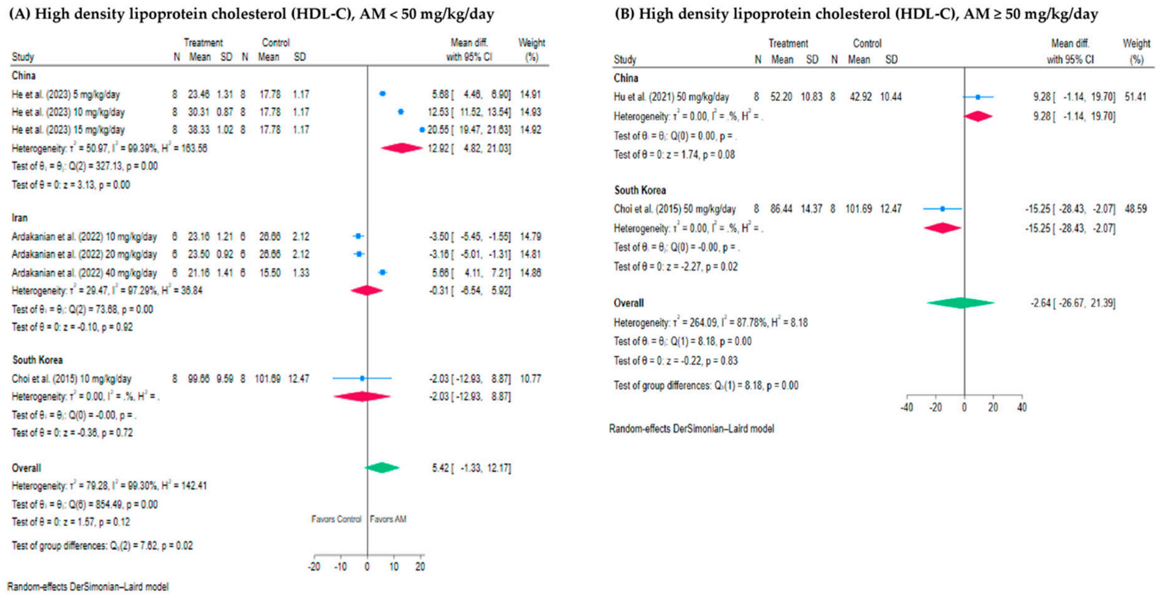

Supplementary Figure S8. Subgroup analysis of the effect of alpha-mangostin (AM) on high density lipoprotein cholesterol (HDL-C) levels by country. (A) AM < 50 mg/kg/day: studies conducted in China, Iran, and South Korea. (B) AM ≥ 50 mg/kg/day: studies conducted in China and South Korea. Each subgroup presents the mean difference in HDL-C levels (mg/dL) between AM-treated and control groups with corresponding 95% confidence intervals (CIs). Squares indicate study effect sizes with size proportional to weight; horizontal lines show 95% CIs; diamonds represent pooled effect sizes with 95% CIs.Analysis was performed using a random-effects model to account for between-study heterogeneity [13, 17, 28, 32].

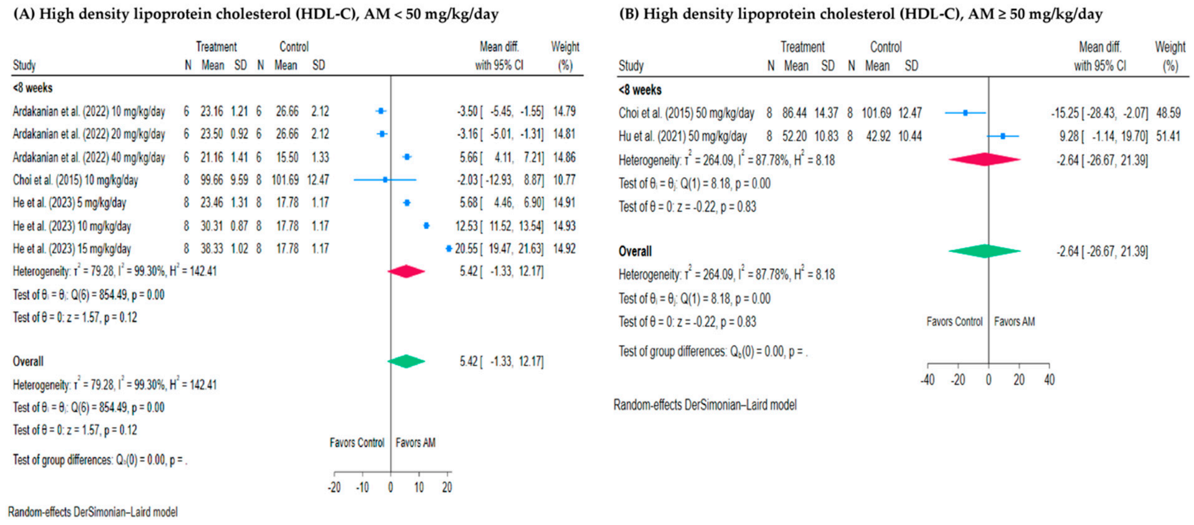

Supplementary Figure S9. Subgroup analysis of the effect of AM on high density lipoprotein cholesterol (HDL-C) levels stratified by treatment duration. (A) Studies using AM < 50 mg/kg/day with treatment durations of < 8 weeks. (B) Studies using AM ≥ 50 mg/kg/day with treatment durations of < 8 weeks. Each forest plot displays the mean difference in HDL-C levels (mg/dL) between AM-treated and control groups with 95% confidence intervals (CIs). Squares indicate study effect sizes with size proportional to weight; horizontal lines show 95% CIs; diamonds represent pooled effect sizes with 95% CIs. Subgroup heterogeneity and overall effects were assessed using a random-effects model [13, 17, 28, 32].
